# Toeplitz and Hankel determinants of logarithmic coefficients for *r*-valent *q*-starlike and *r*-valent *q*-convex functions

**DOI:** 10.1016/j.mex.2025.103463

**Published:** 2025-07-07

**Authors:** Pishtiwan Othman Sabir, Awara Ahmed Ali

**Affiliations:** Department of Mathematics, College of Science, University of Sulaimani, Sulaymaniyah 46001, Iraq

**Keywords:** Holomorphic functions, Logarithmic coefficients, Multivalent functions, *q*-Starlike functions, *q*-Convex functions, Schwarz functions, Subordination, Janowski functions, Fekete-Szegö inequalities, Toeplitz matrices, Hankel determinants

## Abstract

The aim of the present paper is to extend the notions of q-starlikeness and q-convexity to encompass multivalent q-starlikeness and multivalent q-convexity. We systematically introduce and examine subfamilies of r-valently holomorphic functions within the open unit disk D by employing the fractional q-derivative operator, along with the principle of subordination between holomorphic functions.

• The families we define in this paper constitute a generalization of numerous established classes available in existing literature.
• We derive the Fekete-Szegö inequalities for these newly introduced families.
• As a result, we apply these findings to establish bounds for the Toeplitz and Hankel determinants $T_{2,1}\left(\gamma_{u}\right),T_{2,2}\left(\gamma_{u}\right)$ and $H_{2,1}\left(\gamma_{u}\right)$, defined as follows:
  $$T_{2,1}\left(\gamma_{u}\right)=\left|\begin{array}{cc} \gamma_{1} & \gamma_{2}\\ \gamma_{2} & \gamma_{1} \end{array}\right|,\;T_{2,2}\left(\gamma_{u}\right)=\left|\begin{array}{cc} \gamma_{2} & \gamma_{3}\\ \gamma_{3} & \gamma_{2} \end{array}\right|\;\;\text{and}\;\;H_{2,1}\left(\gamma_{u}\right)=\left|\begin{array}{cc} \gamma_{1} & \gamma_{2}\\ \gamma_{2} & \gamma_{3} \end{array}\right|$$
  where $\gamma_{1},\gamma_{2}$, and $\gamma_{3}$ denote the first, second, and third logarithmic coefficients of functions within the family of multivalent q-starlike and multivalent q-convex functions.

## Graphical abstract

**Figure:**
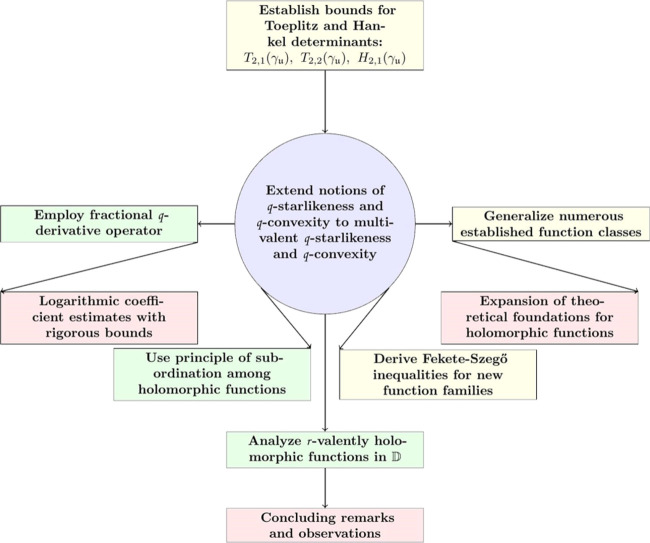

**Specifications table**

**Table:** 

**Subject area**	Mathematics and Statistics
**More specific subject area**	Fractional calculus and analytic functions
**Name of your method**	Differential subordination for r-valent functions
**Name and reference of original method**	Differential Subordination: W.C. Ma, D. Minda, A unified treatment of some special classes of univalent functions, in: Proceedings of the Conference on Complex Analysis (Tianjin, 1992), Internat. Press, Cambridge, MA, 157–169
**Resource availability**	Not applicable

## Background

A significant and fascinating area in complex analysis involves examining the geometric properties of holomorphic functions within D. Properties such as starlikeness and convexity are closely associated with the coefficients of their Taylor-Maclaurin series expansion. Although the graph of a holomorphic function cannot be directly depicted, its image set exhibits a distinct and well-defined geometric structure. The study of univalent functions plays a fundamental role in geometric function theory (GFT). Over the years, this field has expanded significantly, leading to new research directions and numerous important findings and applications. Its foundations are closely linked to Bieberbach's famous conjecture. Bieberbach demonstrated that for any univalent function $u\left(z\right)=z+\sum_{n=2}^{\infty }a_{n}z^{n}$, the bound $|a_{2}|\leq 2$ holds. Durin [1] further conjectured that, in general, the inequality $|a_{n}|\leq n$, n∈N:={1,2,3,⋯}∖{1} holds. The conjecture stimulated further research on the coefficient bounds of univalent functions, leading to significant advancements in GFT.

Multivalent (briefly, r-valent) functions, extend the concept of univalent functions. Many studies have studied subfamilies of holomorphic functions, focusing in particular on different families of r-valent functions. Recent works such as [1] have examined coefficient bounds associated with r-valent functions. Furthermore, neighborhoods of certain r-valent holomorphic functions characterized by negative coefficients have been investigated in [2]. For discussions on Hadamard product (convolution) techniques related to convexity and starlikeness within the context of meromorphic r-valent functions, readers may consult [3]. Additionally, there has been growing interest in studying Taylor-Maclaurin coefficient estimates for r-valent functions, as demonstrated in [[4], [5], [6]].

Quantum calculus (Jackson calculus or briefly, q-calculus) is a mathematical discipline that derives q-analogous results without relying on limits. Unlike classical calculus, where derivatives are defined through the limit process of the difference quotient as the input change approaches zero, q-calculus operates without this limiting procedure. Initially introduced by Jackson [7], this field has since garnered significant interest from researchers in both pure and applied mathematics. Jackson calculus finds applications in multiple fields. For example, within number theory, it introduces concepts such as q-(real and complex numbers). In combinatorics, fundamental ideas include q-(binomial, Taylor, and hypergeometric series). The q-analogues involve the q-(gamma, zeta, beta, exponential, and trigonometric function) in the context of special functions. Significant topics in calculus include q-(fractional, differentiation, and integration). Although substantial progress has been made in this domain, many open problems persist, ensuring that quantum calculus remains an active and evolving field of study. For instance, Silviya and Muthunagai [8] developed new sandwichtype inequalities that incorporate fuzzy differential operators by combining q-calculus with fuzzy logic and studied differential and fuzzy differential sandwich theorems using q-derivative operators. Alqahtani et al. [9] characterized generalized q-convex functions using q-calculus, contributing to the deeper understanding of convexity in quantum calculus. Çağlar et al. [10] examined coefficient bounds for q-starlike functions related to q-Bernoulli numbers, bridging the gap between classical starlike functions and q-calculus. Louati et al. [11] defined and analyzed new subfamilies linked to symmetrical functions and quantum calculus, enriching the theoretical foundations of q-based function analysis. In addition, Lupaş [12] introduced a convolution operator of the q-hypergeometric type and studied strongly differential subordination and superordination by analyzing distortion constraints, and coefficient bounds.

The Fekete-Szegö problem holds significant importance in GFT, as it concerns the determination of bounds for the functional $|a_{3}-\mu a_{2}^{2}|$, where the parameter μ is a real or complex number. Its origins can be traced to the research of Fekete and Szegö [13], who disproved the Littlewood-Paley conjecture. Moreover, the Hankel determinant for univalent functions was initiated by the same authors, play a crucial role in GFT who specifically investigated the determinant

$$H_{2}\left(1\right)=\left|\begin{array}{cc} a_{1} & a_{2}\\ a_{2} & a_{3} \end{array}\right|$$

Subsequent research on these determinants was conducted by Pommerenke [14], and followed by Noonan and Thomas [15]. They generalized the determinant for m,n∈N as follows:

$$H_{m}\left(n\right)=\left|\begin{array}{cccc} a_{n} & a_{n+1} & \cdots & a_{n+m-1}\\ a_{n+1} & a_{n+2} & \cdots & a_{n+m}\\ \vdots & \vdots & \vdots & \vdots \\ a_{n+m-1} & a_{n+m} & \cdots & a_{n+2m-2} \end{array}\right|$$

Hankel determinants have diverse applications, particularly in random matrix theory and the study of orthogonal polynomials. For an in-depth discussion, see, for instance, the recent contributions by Min and Chen [16].

The research presented by Thomas and Halim [17] introduces the symmetric Toeplitz determinant, denoted as $T_{m}\left(n\right)$, is expressed for m,n∈N as follows:

$$T_{m}\left(n\right)=\left|\begin{array}{cccc} a_{n} & a_{n+1} & \cdots & a_{m+n-1}\\ a_{n+1} & a_{n} & \cdots & a_{m+n-2}\\ \vdots & \vdots & \vdots & \vdots \\ a_{m+n-1} & a_{m+n-2} & \cdots & a_{n} \end{array}\right|$$

Toeplitz and Hankel matrices share a close relationship. Toeplitz matrices display identical elements along their main diagonals, whereas Hankel matrices exhibit constant values along the converse diagonals. In a seminal work in 2016, Ye and Lim [18] established that matrices of size m×m across the set of complex values can generally be considered to derive from the product of specific Toeplitz (or Hankel) matrices. The importance of Toeplitz determinants and matrices spans different mathematical concepts, presenting a diverse scope of applications [18]. Furthermore, in both the theoretical and applied realms of mathematics, Toeplitz determinants and Hankel matrices play vital roles, finding applications in fields such as integral equations and analysis, quantum mechanics, signal and image processing, and others [19]. In recent studies, considerable attention has been devoted to exploring interesting properties associated with Teoplitz and Hankel determinants within the realm of holomorphic functions of certain families of convex and starlike functions (see, for example, [[20], [21], [22]] and references therein).

Inspired by the aforementioned works, starlike and convex functions have gained increased prominence in both academic literature and practical applications over the past decade. Based on the rationale provided by the existing research and recognizing the importance of determinants and logarithmic coefficients, this method aims to establish bounds for the Toeplitz and Hankel determinants whose entries consist of logarithmic coefficients.

The motivation for presenting this methodology arises from the necessity to expand the current comprehension of fractional calculus and its diverse applications. This methodology is based on the emerging concept of r-valent (or multivalent) q-analysis, which serves as a broader framework than traditional q-analysis. Specifically, we delve into the analysis of subfamilies of r-valent holomorphic functions, including the well-known family of Janowski functions within D. To achieve this, we employ the fractional q-derivative operator, in accordance with the principle of subordination between holomorphic functions.

Our method demonstrates that the families introduced in this study generalize numerous preexisting families documented in the literature. Each of these recently introduced function families reveals a variety of compelling properties and characteristics, systematically derived and thoroughly examined. The outcomes of this research will furnish non-sharp bounds for the families of multivalent q-starlike and multivalent q-convex functions, along with various subfamilies thereof. Furthermore, we present logarithmic coefficient estimates, sufficient conditions, and the Fekete-Szegö functional, illuminating a specific relationship between coefficients.

## Method details

### Introduction, definitions and motivation

Let A represent the collection of functions defined by

(2.1) $$u\left(z\right)=z+\sum_{n=2}^{\infty }a_{n}z^{n},$$

where each function is holomorphic in D:={z:z∈C and |z|<1}, and the well-known sets C and D are referred to as the set of complex numbers and the open unit disk, respectively. Moreover, let S represent a subcollection of A, comprising functions that exhibit univalence within D. For any function u∈S, we can define the logarithmic coefficients, denoted as $\gamma_{n}\;\left(n\in N\right)$, through the following equation:

(2.2) $$\log\frac{u\left(z\right)}{z}=2\sum_{n=1}^{\infty }\gamma_{n}z^{n}.$$

Insufficient exact information is available concerning these coefficients, which play a significant role in Milin's conjecture as discussed in [23]. The natural conjecture, inspired by the Koebe function with logarithmic coefficients of $\frac{1}{n}$, suggests $|\gamma_{n}|\leq \frac{1}{n}$. However, this conjecture proves to be inaccurate, even in terms of magnitude order (see Duren [1]). In contrast to the coefficients of functions within S, we have identified the sole known bounds for the logarithmic coefficients:

$$\left|\gamma_{1}\right|\leq 1\;\text{and}\;\left|\gamma_{2}\right|\leq \frac{1}{2}+\frac{1}{e^{2}}=0.635\ldots$$

and bounds remain unknown for n∈N∖{1,2}. In the domain of GFT, considerable attention has been directed towards subfamilies associated with convex and starlike functions. A function u within the family S is classified as convex if its image u(D) takes on a convex shape, while it is characterized as starlike when the image u(D) exhibits starlikeness with respect to the origin. The most significant and extensively studied subfamilies within the family S are the starlike functions $S^{*}$ and the convex functions $S^{C}$. Therefore, by definition (see [1]), we can determine for z∈D

(2.3) S*:={u:u∈SandRe(zu′(z)u(z))>0}

and

(2.4) SC:={u:u∈SandRe(1+zu'′(z)u′(z))>0}.

We note that

$$S^{C}\subset S^{*},$$

$$\begin{array}{c} u\left(z\right)\in S^{C}\;\;\text{if}\;\text{and}\;\text{only}\;\text{if}\;\;zu^{\prime }\left(z\right)\in S^{*}, \end{array}$$

and

$$u\left(z\right)\in S^{*}\;\;\text{if}\;\text{and}\;\text{only}\;\text{if}\;\;\underset{0}{\int^{z}}\frac{u\left(s\right)}{s}\mathrm{ds}\in S^{C}.$$

Let H be the collection of holomorphic functions h:D→C that satisfy the conditions h(0)=1 and Re(h(z))>0 for any z∈D, and let Ω denote the set comprising all Schwarz functions. A holomorphic function $u_{1}$ is considered subordinate to another holomorphic function $u_{2}$ (see [24]), if there exists a Schwarz function ω, which is holomorphic within D, and satisfies ω(0)=0 and |ω(z)|<1, such that $u_{1}\left(z\right)=u_{2}\left(\omega (z)\right)$ for any z∈D. It is denoted by $u_{1}≺u_{2}$. Significantly, Ma and Minda [25] have provided a unifying framework for various subfamilies of starlike and convex functions. They formally established

(2.5) $$\begin{array}{c} S^{*}\left(\Phi \right):=\left\{u:u\in S\;\;\text{and}\;\;\frac{zu^{\prime }\left(z\right)}{u\left(z\right)}≺\Phi \left(z\right)\right\} \end{array}$$

and

(2.6) $$\begin{array}{c} S^{C}\left(\Phi \right):=\left\{u:u\in S\;\;\text{and}\;\;1+\frac{zu^{'\prime }\left(z\right)}{u^{\prime }\left(z\right)}≺\Phi \left(z\right)\right\}, \end{array}$$

where $\Phi \in H,\Phi^{\prime }\left(0\right)>0$ and Φ(D) is symmetric about the real axis. A function characterized by this structure is represented by the ensuing series expansion:

(2.7) $$\Phi \left(z\right)=1+J_{1}z+J_{2}z^{2}+J_{3}z^{3}+\cdots ,\;\left(J_{1}>0\;\text{and}\;J_{2},J_{3}\;\text{is}\;\text{any}\;\text{real}\;\text{number}\;\right).$$

A holomorphic function u defined in a domain D∈C is identified as r-valent within D if, for any complex number w, the equation u(z)=w has at most r roots within D (see [26]). This condition implies the existence of a specific complex number $w_{0}$ for which the equation $u\left(z\right)=w_{0}$ possesses precisely r solutions within the domain D. Let A(r) represent the family of functions defined by

(2.8) $$\begin{array}{c} u\left(z\right)=z^{r}+\sum_{n=r+1}^{\infty }a_{n}z^{n},\;\left(r\in N\right), \end{array}$$

which exhibit holomorphicity and r-valency within D. For r=1, the expansion (2.8) coincides with (2.1) of the family A=A(1).

The subfamily of A(r) that includes the collection of univalent functions is denoted by S(r). For each function u∈S(r), we define the following equation:

(2.9) $$F_{u}\left(z\right)=\log\frac{u\left(z\right)}{z^{r}}=2r\sum_{n=1}^{\infty }\gamma_{n}\left(u\right)z^{n},$$

where for all n∈N, the number $\gamma_{n}≔\gamma_{n}\left(u\right)$ is referred to as the logarithmic coefficient of the function u. For r=1, the expansion in (2.9) coincides with (2.2) of the family S=S(1). Comparing corresponding powers of z in (2.9) reveals that

(2.10) $$\gamma_{1}\;=\frac{1}{2r}a_{r+1},\;$$

(2.11) $$\gamma_{2}\;=\frac{1}{2r}\left(a_{r+2}-\frac{1}{2}a_{r+1}^{2}\right),$$

(2.12) $$\gamma_{3}\;=\frac{1}{2r}\left(a_{r+3}-a_{r+1}a_{r+2}+\frac{1}{3}a_{r+1}^{3}\right).$$

Quantum (or q)-analysis serves as a broader framework than conventional analysis, eliminating the need for limit notation. Its initial application was pioneered by Jackson in [27] and [28], marking the inception of q-calculus. Subsequently, diverse applications spanning Mathematics and Physics have been examined, as detailed in [29,30]. In the realm of GFT, where starlikeness and convexity are pivotal, several publications leverage the q-differential operator (see, for example, [[31], [32], [33]]).

To enhance comprehension of the paper, let's review key definitions and concepts related to the q-difference calculus. Throughout the entirety of the paper, we assumed that 0<q<1.

The q-number ${\left[l\right]}_{q}$ (see [34]) is defined as

$${\left[l\right]}_{q}=\left\{ \begin{array}{cc} \sum_{k=0}^{l-1}q^{k}, & \;\text{if}\;\;l\in N,\\ \frac{1-q^{l}}{1-q}, & \;\text{if}\;\;l\in C. \end{array}\right.$$

It is worth mentioning that ${\left[0\right]}_{q}=0$, and as $q\to 1^{-},{\left[l\right]}_{q}\to l$. For $u\left(z\right)=z+\sum_{n=2}^{\infty }a_{n}z^{n}\in A$, the q-derivative operator $D_{q}$ (see [[27], [28]]) is defined as

(2.13) $$\begin{array}{ccc} D_{q}u\left(z\right) & = & \frac{u\left(\mathrm{qz}\right)-u\left(z\right)}{\left(q-1\right)z},\;\left(z\neq 0,q\neq 1\right),\\ & = & 1+\sum_{n=2}^{\infty }{\left[n\right]}_{q}a_{n}z^{n-1}. \end{array}$$

It is evident that as $q\to 1^{-},D_{q}u\left(z\right)\to u^{\prime }\left(z\right)$. We now broaden the application of the q-difference operator to a function u defined by (2.8) within the family A(r) in the subsequent way:

(2.14) $$D_{q}u\left(z\right)\;={\left[r\right]}_{q}z^{r-1}+\sum_{n=r+1}^{\infty }{\left[n\right]}_{q}a_{n}z^{n-1}.$$

Utilizing the definitions provided above and the principle of subordination, we proceed to introduce the following families:

Definition 2.1 A function u, defined by (2.8) and belonging to A(r), is regarded as a member of the family $S_{q}^{*}\left(r,\Phi \right)$ if it satisfies the subsequent subordination criterion:

(2.15) $$\begin{array}{c} S_{q}^{*}\left(r,\Phi \right):=\left\{u:u\in S\left(r\right)\;\text{and}\;\;\frac{zD_{q}u\left(z\right)}{{\left[r\right]}_{q}u\left(z\right)}≺\Phi \left(z\right)\right\}. \end{array}$$

Definition 2.2 A function u, defined by (2.8) and belonging to the set A(r), is recognized as a member of the family $S_{q}^{C}\left(r,\Phi \right)$ if it meets the following subordination criterion:

(2.16) $$\begin{array}{c} S_{q}^{C}\left(r,\Phi \right):=\left\{u:u\in S\left(r\right)\;\text{and}\;\;\frac{D_{q}\left(zD_{q}u\left(z\right)\right)}{{\left[r\right]}_{q}D_{q}u\left(z\right)}≺\Phi \left(z\right)\right\}. \end{array}$$

Remark 2.1 We observe that,

1. For r=1, the families $S_{q}^{*}\left(r,\Phi \right)$ and $S_{q}^{C}\left(r,\Phi \right)$ reduce to the families $S_{q}^{*}\left(\Phi \right)$ and $S_{q}^{C}\left(\Phi \right)$, respectively, which were considered by Seoudy and Aouf [35] and Cetinkaya et al. [36].
2. For $q\to 1^{-}$, the families $S_{q}^{*}\left(r,\Phi \right)$ and $S_{q}^{C}\left(r,\Phi \right)$ reduce to the families $S^{*}\left(r,\Phi \right)$ and $S^{C}\left(r,\Phi \right)$, respectively, which were investigated by Ali et al. [37].
3. For r=1 and $q\to 1^{-}$, the families $S_{q}^{*}\left(r,\Phi \right)$ and $S_{q}^{C}\left(r,\Phi \right)$ reduce to the families $S^{*}\left(\Phi \right)$ and $S^{C}\left(\Phi \right)$, which are specified by (2.5) and (2.6), respectively.

**Fig. 1:**
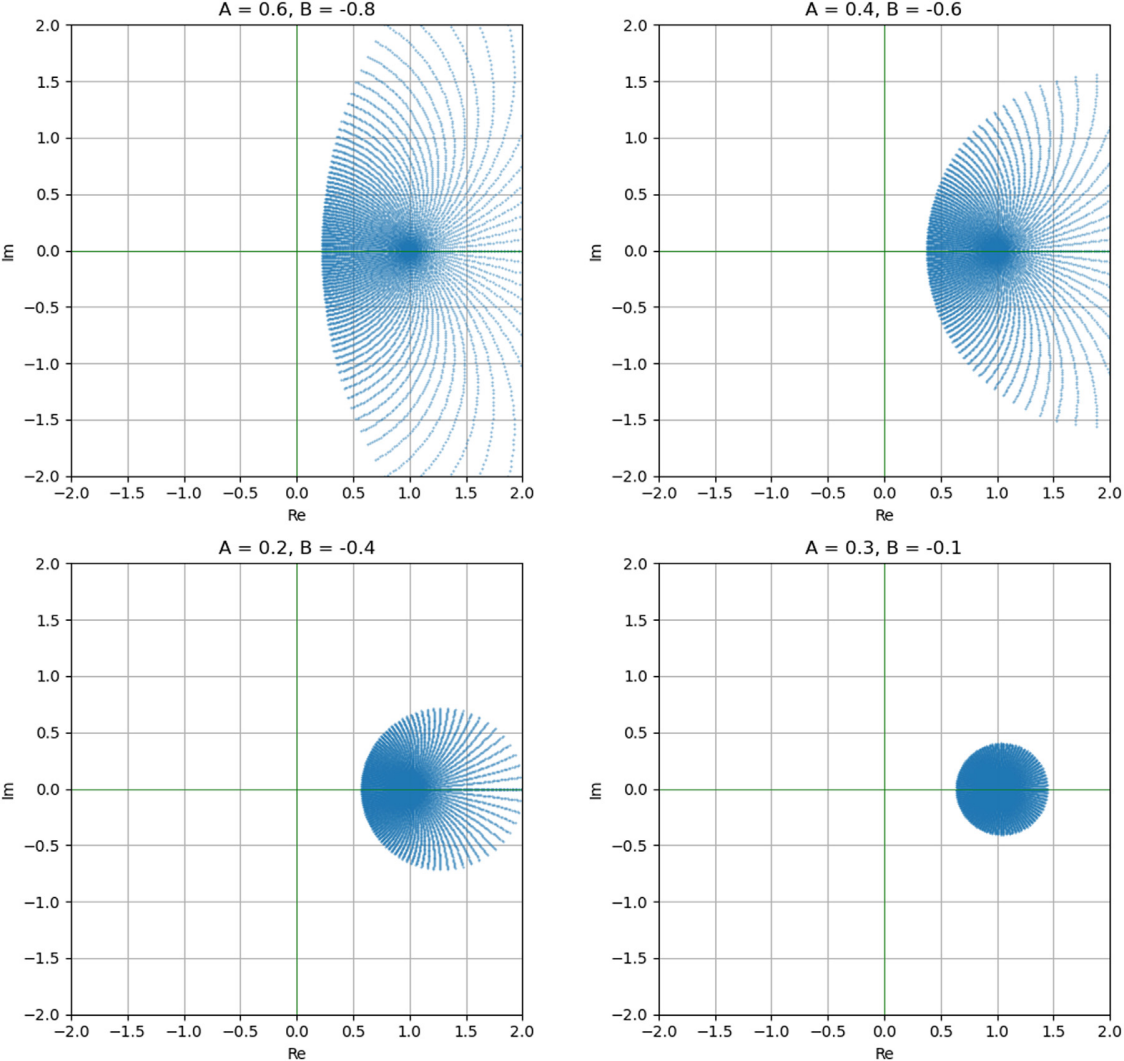
Image of D under Φ(z) defined by (2.17).

If we set the Janowski function (see Fig. 1)

(2.17) $$\Phi \left(z\right)=\frac{1+Az}{1+Bz},\;\left(-1\leq B<A\leq 1\right),$$

then the families $S_{q}^{*}\left(r,\Phi \right)$ and $S_{q}^{C}\left(r,\Phi \right)$ reduces to the families $S_{q}^{*}\left(r,A,B\right)$ and $S_{q}^{C}\left(r,A,B\right)$, respectively, which are defined by assuming that u∈A(r) given by (2.8),

(2.18) $$\begin{array}{c} S_{q}^{*}\left(r,A,B\right):=\left\{u:u\in S\left(r\right)\;\text{and}\;\;\frac{zD_{q}u\left(z\right)}{{\left[r\right]}_{q}u\left(z\right)}≺\frac{1+\mathrm{Az}}{1+\mathrm{Bz}}\right\} \end{array}$$

and

(2.19) $$\begin{array}{c} S_{q}^{C}\left(r,A,B\right):=\left\{u:u\in S\left(r\right)\;\text{and}\;\;\frac{D_{q}\left(zD_{q}u\left(z\right)\right)}{{\left[r\right]}_{q}D_{q}u\left(z\right)}≺\frac{1+\mathrm{Az}}{1+\mathrm{Bz}}\right\}. \end{array}$$

Remark 2.2 We note that,

1. For r=1, the families $S_{q}^{*}\left(r,A,B\right)$ and $S_{q}^{C}\left(r,A,B\right)$ reduce to the families of Janowski q-starlike functions, denoted as $S_{q}^{*}\left[A,B\right]$, and Janowski q-convex functions, denoted as $S_{q}^{C}\left[A,B\right]$, respectively.
2. For $q\to 1^{-}$, the families $S_{q}^{*}\left(r,A,B\right)$ and $S_{q}^{C}\left(r,A,B\right)$ reduce to the families of Janowski r-valently starlike function $S^{*}\left[r,A,B\right]$, and Janowski r-valently convex function $S^{C}\left[r,A,B\right]$, respectively.
3. For r=1 and $q\to 1^{-}$, the families $S_{q}^{*}\left(r,A,B\right)$ and $S_{q}^{C}\left(r,A,B\right)$ reduce to the families of Janowski starlike functions denoted as $S^{*}\left[A,B\right]$ and Janowski convex functions denoted as $S^{C}\left[A,B\right]$, respectively (see [38]).
4. The families $S_{q}^{*}\left(r,A,B\right)$ and $S_{q}^{C}\left(r,A,B\right)$ can be alternatively represented as follows:
  $$S_{q}^{*}\left(r,A,B\right):=\left\{u:u\in S\left(r\right)\;\text{and}\;\;\left|\frac{zD_{q}u\left(z\right)-{\left[r\right]}_{q}u\left(z\right)}{A{\left[r\right]}_{q}u\left(z\right)-\mathrm{Bz}D_{q}u\left(z\right)}\right|<1\right\}$$
  and
  $$S_{q}^{C}\left(r,A,B\right):=\left\{u:u\in S\left(r\right)\;\text{and}\;\;\left|\frac{D_{q}\left(zD_{q}u\left(z\right)\right)-{\left[r\right]}_{q}D_{q}u\left(z\right)}{A{\left[r\right]}_{q}D_{q}u\left(z\right)-BD_{q}\left(zD_{q}u\left(z\right)\right)}\right|<1\right\}.$$

**Fig. 2:**
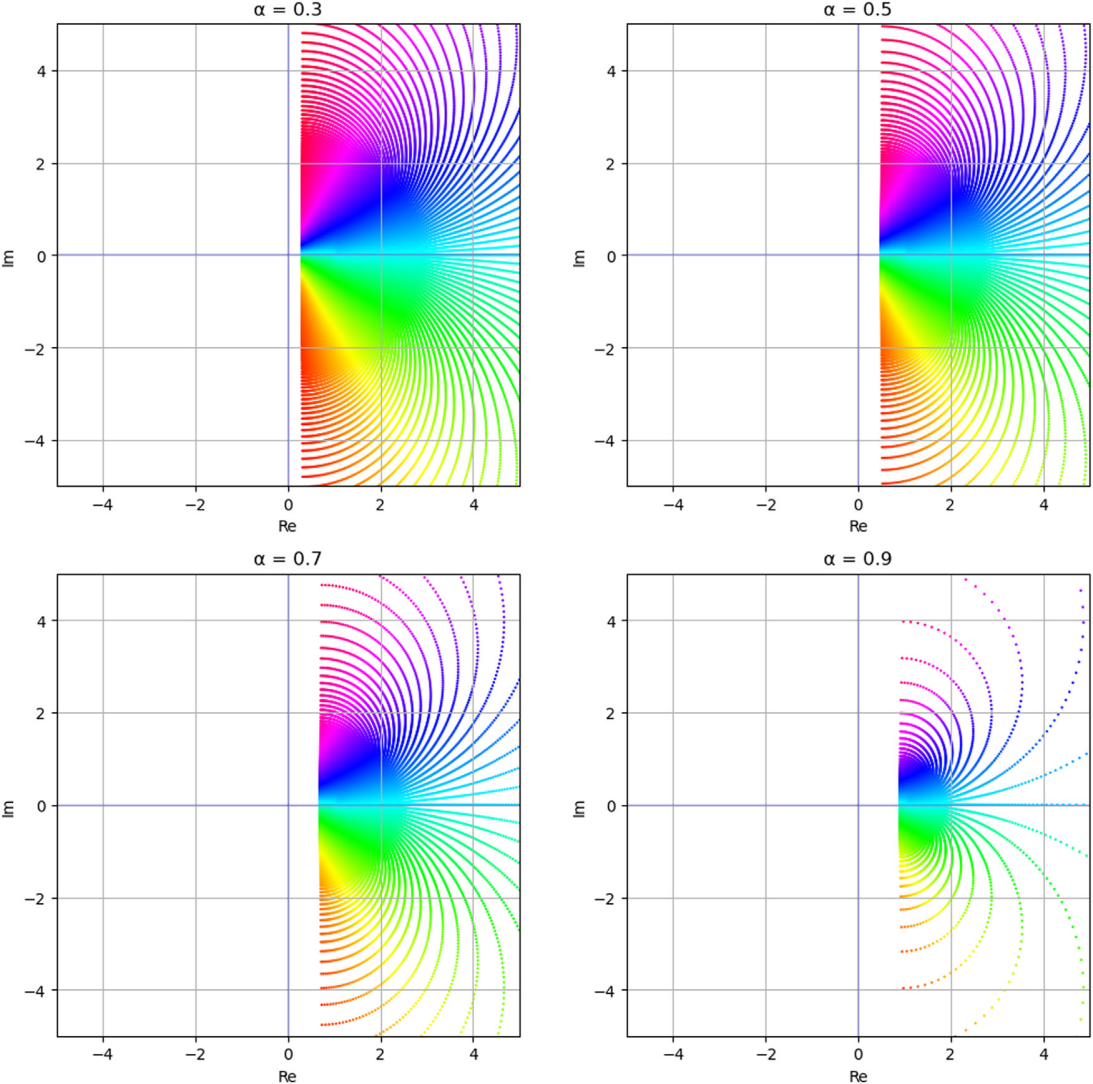
Image of D under Φ(z) defined by (2.20).

If we take (see Fig. 2)

(2.20) $$\Phi \left(z\right)=\frac{1+\left(1-2\alpha \right)z}{1-z},\;\left(0\leq \alpha <1\right),$$

then the families $S_{q}^{*}\left(r,\Phi \right)$ and $S_{q}^{C}\left(r,\Phi \right)$ reduce to the families $S_{q}^{*}\left(r,\alpha \right)$ and $S_{q}^{C}\left(r,\alpha \right)$, respectively, which are defined by assuming that u∈A(r) given by (2.8),

(2.21) Sq*(r,α):={u:u∈S(r)andRe(zDqu(z)[r]qu(z))>α}

and

(2.22) SqC(r,α):={u:u∈S(r)andRe(Dq(zDqu(z))[r]qDqu(z))>α}.

Remark 2.3 We note that,

1. The families $S_{q}^{*}\left(r,\alpha \right)$ and $S_{q}^{C}\left(r,\alpha \right)$ were given recently by Srivastava et al. [39].
2. For r=1, the families $S_{q}^{*}\left(r,\alpha \right)$ and $S_{q}^{C}\left(r,\alpha \right)$ reduce to the families $S_{q}^{*}\left(\alpha \right)$ and $S_{q}^{C}\left(\alpha \right)$, respectively. These families were investigated by Seoudy and Aouf [35].
3. For $q\to 1^{-}$, the families $S_{q}^{*}\left(r,\alpha \right)$ and $S_{q}^{C}\left(r,\alpha \right)$ reduce to the families $S^{*}\left(r,\alpha \right)$ and $S^{C}\left(r,\alpha \right)$, respectively, which were defined by Hayami and Owa [40]
4. For α=0 and r=1, the family $S_{q}^{*}\left(r,\alpha \right)$ reduces to the family $S_{q}^{*}$, which was first introduced by Ismail et al. [41], and the family $S_{q}^{C}\left(r,\alpha \right)$ reduces to the family $S_{q}^{C}$, which was introduced by Ahuja et al. [42].
5. For r=1 and $q\to 1^{-}$, the families $S_{q}^{*}\left(r,\alpha \right)$ and $S_{q}^{C}\left(r,\alpha \right)$ reduce to the well-established families of starlike functions $S^{*}\left(\alpha \right)$ of order α(0≤α<1) and convex functions $S^{C}\left(\alpha \right)$ of order α(0≤α<1), respectively (see Duren [1]).
6. For α=0, r=1 and $q\to 1^{-}$the families $S_{q}^{*}\left(r,\alpha \right)$ and $S_{q}^{C}\left(r,\alpha \right)$ reduce to the families $S^{*}$, defined by (2.3) and $S^{C}$, defined by (2.4), respectively.

**Fig. 3:**
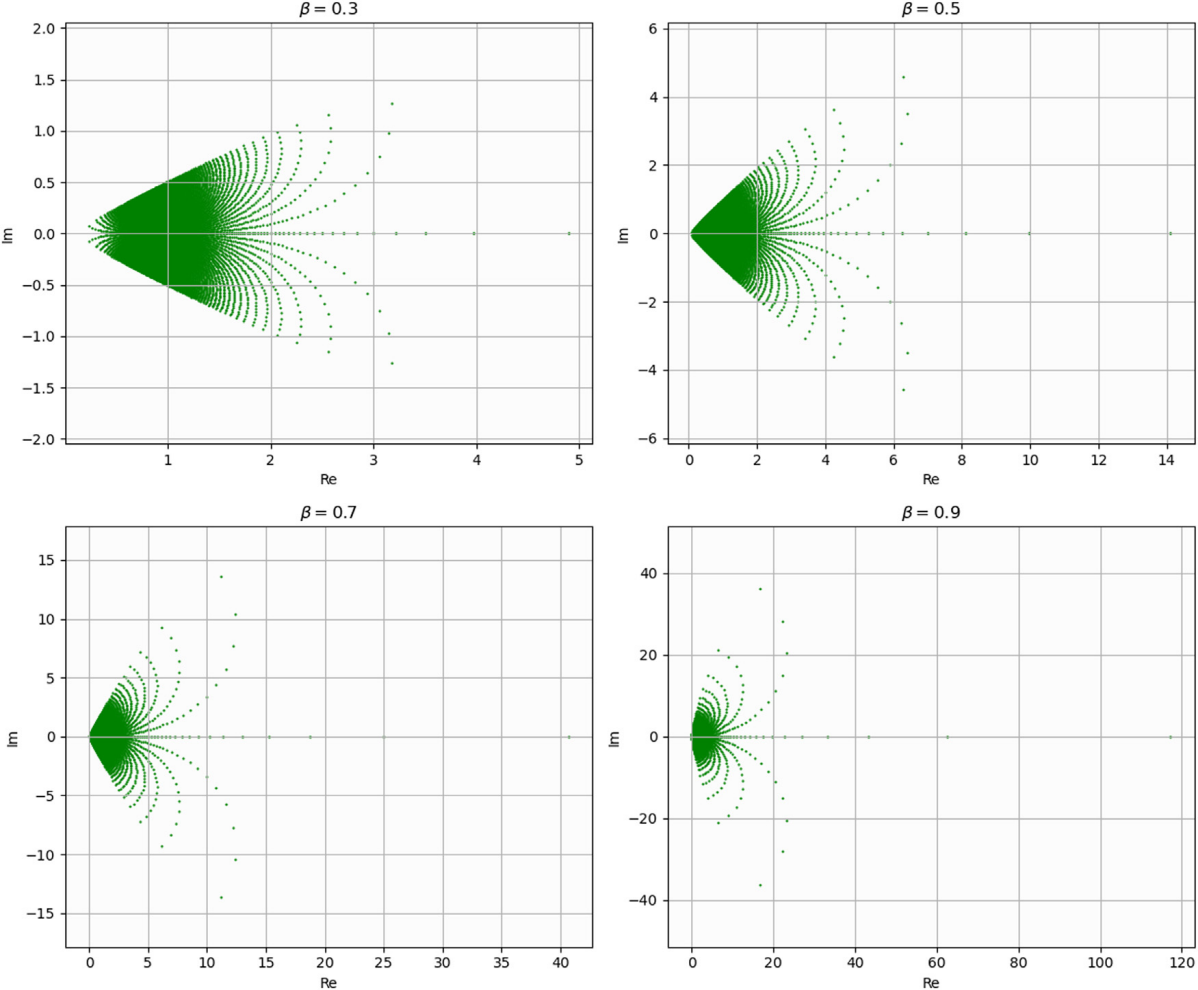
Image of D under Φ(z) defined by (2.23).

If we put (see Fig. 3)

(2.23) $$\Phi \left(z\right)={\left(\frac{1+z}{1-z}\right)}^{\beta },\;\left(0<\beta \leq 1\right),$$

then the families $S_{q}^{*}\left(p,\Phi \right)$ and $S_{q}^{C}\left(p,\Phi \right)$ reduce to the families $S_{q}^{*}\left(r,\beta \right)$ and $S_{q}^{C}\left(r,\beta \right)$, respectively, which are defined by assuming that u∈A(r) given by (2.8),

(2.24) $$\begin{array}{c} S_{q}^{*}\left(r,\beta \right):=\left\{u:u\in S\left(r\right)\;\text{and}\;arg\left(\frac{zD_{q}u\left(z\right)}{{\left[r\right]}_{q}u\left(z\right)}\right)<\frac{\beta \pi }{2}\right\} \end{array}$$

and

(2.25) $$\begin{array}{c} S_{q}^{C}\left(r,\beta \right):=\left\{u:u\in S\left(r\right)\;\text{and}\;arg\left(\frac{D_{q}\left(zD_{q}u\left(z\right)\right)}{{\left[r\right]}_{q}D_{q}u\left(z\right)}\right)<\frac{\beta \pi }{2}\right\}. \end{array}$$

Remark 2.4 We observe that,

1. For r=1, the families $S_{q}^{*}\left(r,\beta \right)$ and $S_{q}^{C}\left(r,\beta \right)$ reduce to the strongly q-starlike and strongly q-convex functions with order β(0<β≤1), respectively.
2. For $q\to 1^{-}$, the families $S_{q}^{*}\left(r,\beta \right)$ and $S_{q}^{C}\left(r,\beta \right)$ reduce to the strongly r-valently starlike and strongly r-valently convex functions with order β(0<β≤1), respectively.
3. For r=1 and $q\to 1^{-}$, the families $S_{q}^{*}\left(r,\beta \right)$ and $S_{q}^{C}\left(r,\beta \right)$ reduce to the families $S^{*}\left(\beta \right)$ and $S^{C}\left(\beta \right)$ of strongly starlike and strongly convex functions with order β(0<β≤1), respectively.

Several recent studies have concentrated on establishing the upper bounds for Toeplitz and Hankel determinants connected with functions in the set A. In a recent contribution, Kowalczyk and Lecko [43] presented the concept of the Hankel determinant, wherein the entries comprised the logarithmic coefficients of functions within the set A. Their research involved a detailed exploration of precise estimates for second Hankel determinants of logarithmic coefficients, focusing specifically on functions within the collections $S^{*}$ and $S^{C}$. This investigation was further extended to the families $S^{*}\left(\alpha \right)$ and $S^{C}\left(\alpha \right)$ by the same authors in [44]. More recently, Sabir in [45] extensively studies sharp bounds on Toeplitz determinants of logarithmic coefficients for convex and starlike functions associated with bilinear transformations, while Biswas in [46] has investigated the sharp bounds of the second Hankel determinant of logarithmic coefficients for functions within specific classes of univalent functions (see also [[47], [48]] and references cited therein).

The Toeplitz and Hankel determinants $H_{m,n}\left(\gamma_{u}\right)$ and $T_{m,n}\left(\gamma_{u}\right)$ of logarithmic coefficients of univalent functions is defined for m,n∈N as follows:

$$T_{m,n}\left(\gamma_{u}\right)=\left|\begin{array}{cccc} \gamma_{n} & \gamma_{n+1} & \cdots & \gamma_{m+n-1}\\ \gamma_{n+1} & \gamma_{n} & \cdots & \gamma_{m+n-2}\\ \vdots & \vdots & \vdots & \vdots \\ \gamma_{m+n-1} & \gamma_{m+n-2} & \cdots & \gamma_{n} \end{array}\right|$$

and

$H_{m,n}\left(\gamma_{u}\right)=\left|\begin{array}{cccc} \gamma_{n} & \gamma_{n+1} & \cdots & \gamma_{m+n-1}\\ \gamma_{n+1} & \gamma_{n+2} & \cdots & \gamma_{m+n}\\ \vdots & \vdots & \vdots & \vdots \\ \gamma_{m+n-1} & \gamma_{m+n} & \cdots & \gamma_{2(m-1)+n} \end{array}\right|.$

Consequently, we obtain

(2.26) $$T_{2,1}\left(\gamma_{u}\right)=\gamma_{1}^{2}-\gamma_{2}^{2},T_{2,2}\left(\gamma_{u}\right)=\gamma_{2}^{2}-\gamma_{3}^{2}\;\text{and}\;H_{2,1}\left(\gamma_{u}\right)=\gamma_{1}\gamma_{3}-\gamma_{2}^{2}.$$

Motivated by the existing research and recognizing the significance of determinants and logarithmic coefficients, this study aims to establish bounds for the Toeplitz and Hankel determinants whose entries consist of logarithmic coefficients. We delve into the analysis of specific subfamilies of r-valent holomorphic functions, including the well-known family of Janowski functions within D. To achieve this, we employ the fractional q-derivative operator, in accordance with the principle of subordination between holomorphic functions. Our findings will demonstrate that the families introduced in this paper generalize many preexisting families documented in the literature. Each of these recently introduced function families reveals a variety of compelling properties and characteristics, systematically derived and thoroughly examined. The outcomes of this research will furnish non-sharp bounds for the families of multivalent q-starlike and multivalent q-convex functions, along with various subfamilies thereof. Furthermore, we present logarithmic coefficient estimates, sufficient conditions, and the Fekete-Szegö functional, illuminating a specific relationship between coefficients.

For the sake of simplicity throughout the next four sections, we assume that $\vartheta_{1},\vartheta_{2},\vartheta_{3}$, and $\vartheta_{4}$ represent ${\left[r\right]}_{q},{\left[r+1\right]}_{q},{\left[r+2\right]}_{q}$, and ${\left[r+3\right]}_{q}$, respectively.

**A set of lemmas**

By utilizing the lemmas presented below, we will demonstrate our primary findings.

Lemma 3.1 [ 25,37] *If* $\omega \left(z\right)=\sum_{n=1}^{\infty }L_{n}z^{n}\in \Omega$, *then*

$$\left|L_{2}-\eta L_{1}^{2}\right|\leq \left\{ \begin{array}{cc} -\eta , & \;\text{if}\;\eta \leq -1,\\ 1, & \;\text{if}\;-1\leq \eta \leq 1,\\ \eta , & \;\text{if}\;\eta \geq 1. \end{array}\right.$$

Lemma 3.2 [ 49] *If* $\omega \left(z\right)=\sum_{n=1}^{\infty }L_{n}z^{n}\in \Omega$ *and* $\left(\rho ,\delta \right)\in \cup_{i=1}^{5}D_{i}$, *then*

$$\left|L_{3}+\rho L_{1}L_{2}+\delta L_{3}\right|\leq \left|\delta \right|,$$

where

$$\begin{array}{cc} & D_{1}=\left\{\left(\rho ,\delta \right):\left|\rho \right|\leq \frac{1}{2},\;\delta \leq -1\right\},\\ & D_{2}=\left\{\left(\rho ,\delta \right):\left|\rho \right|\geq \frac{1}{2},\;\delta \leq -\frac{2}{3}\left(\left|\rho \right|+1\right)\right\},\\ & D_{3}=\left\{\left(\rho ,\delta \right):\left|\rho \right|\leq 2,\;\delta \geq 1\right\},\\ & D_{4}=\left\{\left(\rho ,\delta \right):2\leq \left|\rho \right|\leq 4,\;\delta \geq \frac{1}{12}\left(\rho^{2}+8\right)\right\},\\ & D_{5}=\left\{\left(\rho ,\delta \right):\left|\rho \right|\geq 4,\;\delta \geq \frac{2}{3}\left(\left|\rho \right|-1\right)\right\}. \end{array}$$

The Fekete-Szegö functional for the family $S_{q}^{*}\left(r,\Phi \right)$ is provided in the next lemma.

Lemma 3.3 *If*u*belongs to*$S_{q}^{*}\left(r,\Phi \right)$*, with*Φ(z)*given by*(2.7)*, then*

$$\left|a_{r+2}-\lambda a_{r+1}^{2}\right|\leq \left\{ \begin{array}{cc} \frac{\vartheta_{1}J_{2}}{\vartheta_{3}-\vartheta_{1}}+\frac{\vartheta_{1}^{2}J_{1}^{2}}{\left(\vartheta_{3}-\vartheta_{1}\right)\left(\vartheta_{2}-\vartheta_{1}\right)}-\frac{\lambda \vartheta_{1}^{2}J_{1}^{2}}{{\left(\vartheta_{2}-\vartheta_{1}\right)}^{2}}, & \;\text{if}\;\lambda \leq \tau_{1},\\ \frac{\vartheta_{1}J_{1}}{\vartheta_{3}-\vartheta_{1}}, & \;\text{if}\;\tau_{1}\leq \lambda \leq \tau_{2},\\ \frac{\lambda \vartheta_{1}^{2}J_{1}^{2}}{{\left(\vartheta_{2}-\vartheta_{1}\right)}^{2}}-\frac{\vartheta_{1}^{2}J_{1}^{2}}{\left(\vartheta_{3}-\vartheta_{1}\right)\left(\vartheta_{2}-\vartheta_{1}\right)}-\frac{\vartheta_{1}J_{2}}{\vartheta_{3}-\vartheta_{1}}, & \;\text{if}\;\lambda \geq \tau_{2}, \end{array}\right.$$

where

$$\tau_{1}≔\frac{\vartheta_{2}-\vartheta_{1}}{\vartheta_{3}-\vartheta_{1}}-\frac{{\left(\vartheta_{2}-\vartheta_{1}\right)}^{2}\left(J_{1}-J_{2}\right)}{\vartheta_{1}\left(\vartheta_{3}-\vartheta_{1}\right)J_{1}^{2}}$$

and

$$\tau_{2}≔\frac{\vartheta_{2}-\vartheta_{1}}{\vartheta_{3}-\vartheta_{1}}+\frac{{\left(\vartheta_{2}-\vartheta_{1}\right)}^{2}\left(J_{1}+J_{2}\right)}{\vartheta_{1}\left(\vartheta_{3}-\vartheta_{1}\right)J_{1}^{2}}.$$

Proof Let $u\in S_{q}^{*}\left(r,\Phi \right)$ be of the form (2.8). Subsequently, a Schwarz function exists, denoted as $\omega \left(z\right)=\;\sum_{n=1}^{\infty }L_{n}z^{n}$, such that

(3.1) $$\begin{array}{c} \frac{zD_{q}u\left(z\right)}{\vartheta_{1}u\left(z\right)}=\Phi \left(\omega \left(z\right)\right),\;z\in D. \end{array}$$

By expanding the Taylor series of u,Φ and ω, we derive

(3.2) $$\begin{array}{ccc} \frac{zD_{q}u\left(z\right)}{\vartheta_{1}u\left(z\right)} & = & 1+\frac{1}{\vartheta_{1}}\left(\vartheta_{2}-\vartheta_{1}\right)a_{r+1}z+\frac{1}{\vartheta_{1}}\left(\left(\vartheta_{3}-\vartheta_{1}\right)a_{r+2}-\left(\vartheta_{2}-\vartheta_{1}\right)a_{r+1}^{2}\right)z^{2}\\ & & +\;\frac{1}{\vartheta_{1}}\left(\left(\vartheta_{4}-\vartheta_{1}\right)a_{r+3}-\left(\vartheta_{3}+\vartheta_{2}-2\vartheta_{1}\right)a_{r+1}a_{r+2}+\left(\vartheta_{2}-\vartheta_{1}\right)a_{r+1}^{3}\right)z^{3}+\cdots \end{array}$$

and

(3.3) $$\Phi \left(\omega \left(z\right)\right)=1+J_{1}L_{1}z+\left(J_{1}L_{2}+J_{2}L_{1}^{2}\right)z^{2}+\left(J_{1}L_{3}+2J_{2}L_{1}L_{2}+J_{3}L_{1}^{3}\right)z^{3}+\cdots .$$

Through a comparison of corresponding powers in (3.1) and utilizing (3.2) and (3.3), we can represent the coefficients $a_{r+1},a_{r+2}$, and $a_{r+3}$ as

(3.4) $$a_{r+1}=\frac{\vartheta_{1}J_{1}L_{1}}{\vartheta_{2}-\vartheta_{1}},$$

(3.5) $$a_{r+2}=\frac{\vartheta_{1}}{\vartheta_{3}-\vartheta_{1}}\left(\frac{\vartheta_{1}J_{1}^{2}L_{1}^{2}}{\vartheta_{2}-\vartheta_{1}}+J_{2}L_{1}^{2}+J_{1}L_{2}\right),$$

and

(3.6) $$\begin{array}{c} a_{r+3}=\frac{\vartheta_{1}^{2}}{\left(\vartheta_{4}-\vartheta_{1}\right)\left(\vartheta_{3}-\vartheta_{1}\right)\left(\vartheta_{2}-\vartheta_{1}\right)}\left(\vartheta_{1}J_{1}^{3}L_{1}^{3}+\left(\vartheta_{3}+\vartheta_{2}-2\vartheta_{1}\right)\times \left(J_{1}J_{2}L_{1}^{3}+J_{1}^{2}L_{1}L_{2}\right)\right) \end{array}+\frac{\vartheta_{1}}{\left(\vartheta_{4}-\vartheta_{1}\right)}\left(J_{3}L_{1}^{3}+2J_{2}L_{1}L_{2}+J_{1}L_{3}\right).$$

Using (3.4) and (3.5), we have

(3.7) $$a_{r+2}-\lambda a_{r+1}^{2}=\frac{\vartheta_{1}J_{1}}{\vartheta_{3}-\vartheta_{1}}\left[L_{2}-\xi L_{1}^{2}\right],$$

where

$$\xi ≔\frac{\vartheta_{1}J_{1}}{\vartheta_{2}-\vartheta_{1}}\left(\frac{\lambda \left(\vartheta_{3}-\vartheta_{1}\right)}{\vartheta_{2}-\vartheta_{1}}-1\right)-\frac{J_{2}}{J_{1}}.$$

The intended outcome is achieved by employing Lemma 3.1 to Equation (3.7).

In the next Lemma, we compute the Fekete-Szegö functional for the family $S_{q}^{C}\left(r,\Phi \right)$.

Lemma 3.4 *Let*Φ(z)*be given by*(2.7)*, and let*$u\in S_{q}^{C}\left(r,\Phi \right)$*. Then*

$$\left|a_{r+2}-\lambda a_{r+1}^{2}\right|\leq \left\{ \begin{array}{cc} \frac{\vartheta_{1}^{2}J_{2}}{\vartheta_{3}\left(\vartheta_{3}-\vartheta_{1}\right)}+\frac{\vartheta_{1}^{3}J_{1}^{2}}{\vartheta_{3}\left(\vartheta_{3}-\vartheta_{1}\right)\left(\vartheta_{2}-\vartheta_{1}\right)}-\frac{\lambda \vartheta_{1}^{4}J_{1}^{2}}{\vartheta_{2}^{2}{\left(\vartheta_{2}-\vartheta_{1}\right)}^{2}}, & \;\text{if}\;\lambda \leq \sigma_{1},\\ \frac{\vartheta_{1}^{2}J_{1}}{\vartheta_{3}\left(\vartheta_{3}-\vartheta_{1}\right)}, & \;\text{if}\;\sigma_{1}\leq \lambda \leq \sigma_{2},\\ \frac{\lambda \vartheta_{1}^{4}J_{1}^{2}}{\vartheta_{2}^{2}{\left(\vartheta_{2}-\vartheta_{1}\right)}^{2}}-\frac{\vartheta_{1}^{2}J_{2}}{\vartheta_{3}\left(\vartheta_{3}-\vartheta_{1}\right)}-\frac{\vartheta_{1}^{3}J_{1}^{2}}{\vartheta_{3}\left(\vartheta_{3}-\vartheta_{1}\right)\left(\vartheta_{2}-\vartheta_{1}\right)}, & \;\text{if}\;\lambda \geq \sigma_{2}, \end{array}\right.$$

where

$$\sigma_{1}≔\frac{\vartheta_{2}^{2}\left(\vartheta_{2}-\vartheta_{1}\right)}{\vartheta_{1}\vartheta_{3}\left(\vartheta_{3}-\vartheta_{1}\right)}-\frac{\vartheta_{2}^{2}{\left(\vartheta_{2}-\vartheta_{1}\right)}^{2}\left(J_{1}-J_{2}\right)}{\vartheta_{1}^{2}\vartheta_{3}\left(\vartheta_{3}-\vartheta_{1}\right)J_{1}^{2}}$$

and

$$\sigma_{2}≔\frac{\vartheta_{2}^{2}\left(\vartheta_{2}-\vartheta_{1}\right)}{\vartheta_{1}\vartheta_{3}\left(\vartheta_{3}-\vartheta_{1}\right)}+\frac{\vartheta_{2}^{2}{\left(\vartheta_{2}-\vartheta_{1}\right)}^{2}\left(J_{1}+J_{2}\right)}{\vartheta_{1}^{2}\vartheta_{3}\left(\vartheta_{3}-\vartheta_{1}\right)J_{1}^{2}}.$$

Proof Let $u\in S_{q}^{C}\left(r,\Phi \right)$ be of the form (2.8). Subsequently, a Schwarz function exists, denoted as $\omega \left(z\right)=\;\sum_{n=1}^{\infty }L_{n}z^{n}$, such that

(3.8) $$\begin{array}{c} \frac{D_{q}\left(zD_{q}u\left(z\right)\right)}{\vartheta_{1}D_{q}u\left(z\right)}=\Phi \left(\omega \left(z\right)\right),\;z\in D. \end{array}$$

Using the Taylor series expansion of u, we derive

(3.9) $$\begin{array}{ccc} & & \frac{D_{q}\left(zD_{q}u\left(z\right)\right)}{\vartheta_{1}D_{q}u\left(z\right)}\;=1+\frac{\vartheta_{2}}{\vartheta_{1}}\left(\vartheta_{2}-\vartheta_{1}\right)a_{r+1}z+\left(\frac{\vartheta_{3}}{\vartheta_{1}^{2}}\left(\vartheta_{3}-\vartheta_{1}\right)a_{r+2}-\frac{\vartheta_{2}^{2}}{\vartheta_{1}^{3}}\left(\vartheta_{2}-\vartheta_{1}\right)a_{r+1}^{2}\right)z^{2}\\ & & +\;\left(\frac{\vartheta_{4}}{\vartheta_{1}^{2}}\left(\vartheta_{4}-\vartheta_{1}\right)a_{r+3}-\frac{\vartheta_{3}\vartheta_{2}}{\vartheta_{1}^{3}}\left(\vartheta_{3}+\vartheta_{2}-2\vartheta_{1}\right)a_{r+1}a_{r+2}+\frac{\vartheta_{2}^{3}}{\vartheta_{1}^{4}}\left(\vartheta_{2}-\vartheta_{1}\right)a_{r+1}\right)z^{3}+\ldots .\; \end{array}$$

Through a comparison of equivalent powers in (3.8) with the aid of (3.9) and (3.3), we can represent the coefficients $a_{r+1},a_{r+2}$ and $a_{r+3}$ in the following manner:

(3.10) $$a_{r+1}=\frac{\vartheta_{1}^{2}J_{1}L_{1}}{\vartheta_{2}\left(\vartheta_{2}-\vartheta_{1}\right)},$$

(3.11) $$a_{r+2}=\frac{\vartheta_{1}^{2}}{\vartheta_{3}\left(\vartheta_{3}-\vartheta_{1}\right)}\left(\frac{\vartheta_{1}J_{1}^{2}L_{1}^{2}}{\vartheta_{2}-\vartheta_{1}}+J_{2}L_{1}^{2}+J_{1}L_{2}\right),$$

and

(3.12) $$\begin{array}{ccc} a_{r+3} & = & \frac{\vartheta_{1}^{2}}{\vartheta_{4}\left(\vartheta_{4}-\vartheta_{1}\right)}[\frac{\vartheta_{1}^{2}J_{1}^{3}L_{1}^{3}}{{\left(\vartheta_{2}-\vartheta_{1}\right)}^{2}}\left(\frac{\vartheta_{3}+\vartheta_{2}-2\vartheta_{1}}{\vartheta_{3}-\vartheta_{1}}-1\right)+J_{3}L_{1}^{3}+2J_{2}L_{1}L_{2}+J_{1}L_{3}\\ & & \;\;\;\;+\frac{\vartheta_{1}\left(\vartheta_{3}+\vartheta_{2}-2\vartheta_{1}\right)}{\left(\vartheta_{3}-\vartheta_{1}\right)\left(\vartheta_{2}-\vartheta_{1}\right)}\left(J_{1}J_{2}L_{1}^{3}+J_{1}^{2}L_{1}L_{2}\right)]. \end{array}$$

Using (3.10) and (3.11), we have

(3.13) $$a_{r+2}-\lambda a_{r+1}^{2}=\frac{\vartheta_{1}^{2}J_{1}}{\vartheta_{3}\left(\vartheta_{3}-\vartheta_{1}\right)}\left[L_{2}-\chi L_{1}^{2}\right],$$

where

$$\chi ≔\frac{\vartheta_{1}J_{1}}{\vartheta_{2}-\vartheta_{1}}\left(\frac{\lambda \vartheta_{1}\vartheta_{3}\left(\vartheta_{3}-\vartheta_{1}\right)}{\vartheta_{2}^{2}\left(\vartheta_{2}-\vartheta_{1}\right)}-1\right)-\frac{J_{2}}{J_{1}}.$$

The desired outcome is achieved by applying Lemma 3.1 to (3.13).

**Main results**

In the theorems that follow, we determine logarithmic coefficient estimates for functions in $S_{q}^{*}\left(r,\Phi \right)$ and $S_{q}^{C}\left(r,\Phi \right)$.

Theorem 4.1 *If*u*belongs to*$S_{q}^{*}\left(r,\Phi \right)$*, with*Φ(z)*given by*(2.7)*, then*

$$\left|T_{2,1}\left(\gamma_{u}\right)\right|\leq \left\{ \begin{array}{cc} \frac{\vartheta_{1}^{2}J_{1}^{2}}{4r^{2}}\left[\frac{1}{{\left(\vartheta_{2}-\vartheta_{1}\right)}^{2}}+\frac{1}{\vartheta_{3}-\vartheta_{1}}\right], & \;\text{if}\;\left|t\right|<J_{1},\\ \frac{\vartheta_{1}^{2}J_{1}^{2}}{4r^{2}{\left(\vartheta_{2}-\vartheta_{1}\right)}^{2}}\left[1+\vartheta_{1}^{2}{\left(\frac{J_{1}}{2\left(\vartheta_{2}-\vartheta_{1}\right)}-\frac{J_{1}^{2}+\left(\vartheta_{2}-\vartheta_{1}\right)J_{2}}{\left(\vartheta_{3}-\vartheta_{1}\right)J_{1}}\right)}^{2}\right], & \;\text{if}\;\left|t\right|\geq J_{1}, \end{array}\right.$$

where

(4.1) $$t≔\frac{\vartheta_{1}\left(\vartheta_{3}-\vartheta_{1}\right)J_{1}^{2}}{2{\left(\vartheta_{2}-\vartheta_{1}\right)}^{2}}-\frac{\vartheta_{1}J_{1}^{2}}{\vartheta_{2}-\vartheta_{1}}-J_{2}.$$

Proof By applying the bound $|L_{n}|\leq 1$ on (3.4), we get

(4.2) $$\left|a_{r+1}\right|\leq \frac{\vartheta_{1}J_{1}}{\vartheta_{2}-\vartheta_{1}}.$$

Also, Lemma 3.3 yields that

(4.3) $$\begin{array}{c} {\left|a_{r+2}-\frac{1}{2}a_{r+1}^{2}\right|}^{2}\leq \left\{ \begin{array}{cc} \frac{\vartheta_{1}^{2}J_{1}^{2}}{{\left(\vartheta_{3}-\vartheta_{1}\right)}^{2}}, & \;\text{if}\;\left|t\right|<J_{1},\\ \frac{{\left(\vartheta_{1}^{2}\left(\vartheta_{3}-2\vartheta_{2}+\vartheta_{1}\right)J_{1}^{2}-2\vartheta_{1}{\left(\vartheta_{2}-\vartheta_{1}\right)}^{2}J_{2}\right)}^{2}}{4{\left(\vartheta_{3}-\vartheta_{1}\right)}^{2}{\left(\vartheta_{2}-\vartheta_{1}\right)}^{4}}, & \;\text{if}\;\left|t\right|\geq J_{1}, \end{array}\right. \end{array}$$

where t is defined by (4.1). From (2.10) and (2.11), we obtain

(4.4) $$\left|\gamma_{1}^{2}-\gamma_{2}^{2}\right|=\frac{1}{4r^{2}}\left|a_{r+1}^{2}-{\left(a_{r+2}-\frac{1}{2}a_{r+1}^{2}\right)}^{2}\right|\leq \frac{1}{4r^{2}}\left({\left|a_{r+1}\right|}^{2}+{\left|a_{r+2}-\frac{1}{2}a_{r+1}^{2}\right|}^{2}\right).$$

The required bound follows from (2.26) and (4.4) by using the bounds from (4.2) and (4.3).

Theorem 4.2 *Let*Φ(z)*be given by*(2.7)*, and let*$u\in S^{C}\left(r,\Phi \right)$*. Then*

$$\left|T_{2,1}\left(\gamma_{u}\right)\right|\leq \left\{ \begin{array}{cc} \frac{\vartheta_{1}^{4}J_{1}^{2}}{4r^{2}}\left[\frac{1}{\vartheta_{3}^{2}{\left(\vartheta_{3}-\vartheta_{1}\right)}^{2}}+\frac{1}{\vartheta_{2}^{2}{\left(\vartheta_{2}-\vartheta_{1}\right)}^{2}}\right], & \;\text{if}\;\left|\varepsilon \right|<J_{1},\\ \frac{\vartheta_{1}^{4}J_{1}^{2}}{4r^{2}\vartheta_{3}^{2}{\left(\vartheta_{3}-\vartheta_{1}\right)}^{2}}\left[1+{\left(\frac{\vartheta_{1}^{2}\vartheta_{3}\left(\vartheta_{3}-\vartheta_{1}\right)J_{1}}{2\vartheta_{2}^{2}{\left(\vartheta_{2}-\vartheta_{1}\right)}^{2}}-\frac{\vartheta_{1}J_{1}^{2}+\left(\vartheta_{2}-\vartheta_{1}\right)J_{2}}{\left(\vartheta_{2}-\vartheta_{1}\right)J_{1}}\right)}^{2}\right], & \;\text{if}\;\left|\varepsilon \right|\geq J_{1}, \end{array}\right.$$

where

(4.5) $$\varepsilon ≔\frac{\vartheta_{1}^{2}\vartheta_{3}\left(\vartheta_{3}-\vartheta_{1}\right)J_{1}^{2}}{2\vartheta_{2}^{2}{\left(\vartheta_{2}-\vartheta_{1}\right)}^{2}}-\frac{\vartheta_{1}J_{1}^{2}+\left(\vartheta_{2}-\vartheta_{1}\right)J_{2}}{\vartheta_{2}-\vartheta_{1}}.$$

Proof Applying the bound $|L_{n}|\leq 1$ on (3.10), we get

(4.6) $$\left|a_{r+1}\right|\leq \frac{\vartheta_{1}^{2}J_{1}}{\vartheta_{2}\left(\vartheta_{2}-\vartheta_{1}\right)}.$$

Also, Lemma 3.4 yields that

(4.7) $${\left|a_{r+2}-\frac{1}{2}a_{r+1}^{2}\right|}^{2}\leq \left\{ \begin{array}{cc} \frac{\vartheta_{1}^{4}J_{1}^{2}}{\vartheta_{3}^{2}{\left(\vartheta_{3}-\vartheta_{1}\right)}^{2}}, & \;\text{if}\;\left|\varepsilon \right|<J_{1},\\ \frac{\vartheta_{1}^{4}J_{1}^{2}}{\vartheta_{3}^{2}{\left(\vartheta_{3}-\vartheta_{1}\right)}^{2}}{\left[\frac{\vartheta_{1}J_{1}}{\vartheta_{2}-\vartheta_{1}}\left(\frac{\vartheta_{1}\vartheta_{3}\left(\vartheta_{3}-\vartheta_{1}\right)}{2\vartheta_{2}^{2}\left(\vartheta_{2}-\vartheta_{1}\right)}-1\right)-\frac{J_{2}}{J_{1}}\right]}^{2}, & \;\text{if}\;\left|\varepsilon \right|\geq J_{1}, \end{array}\right.$$

where ε is defined by (4.5). The required bound follows from (2.26) and (4.4) by using the bounds from (4.6) and (4.7).

Theorem 4.3 *Let*Φ(z)*be given by*(2.7)*, and let*u*belong to*$S_{q}^{*}\left(r,\Phi \right)$*. If*$\left(\rho_{1},\delta_{1}\right)\in \cup_{i=1}^{5}D_{i}$*holds, then*

$$\left|T_{2,2}\left(\gamma_{u}\right)\right|\leq \left\{ \begin{array}{cc} \frac{\vartheta_{1}^{2}J_{1}^{2}}{4r^{2}{\left(\vartheta_{3}-\vartheta_{1}\right)}^{2}}\left[1+{\left(\Lambda_{1}\left(J_{1},J_{3}\right)-\Lambda_{2}\left(J_{1},J_{2}\right)\right)}^{2}\right], & \;\text{if}\;\left|t\right|<J_{1},\\ \frac{\vartheta_{1}^{2}J_{1}^{2}}{4r^{2}{\left(\vartheta_{3}-\vartheta_{1}\right)}^{2}{\left(\vartheta_{2}-\vartheta_{1}\right)}^{2}}\left[\Lambda_{3}^{2}\left(J_{1},J_{2}\right)+{\left(\Lambda_{4}\left(J_{1},J_{3}\right)-\Lambda_{5}\left(J_{1},J_{2}\right)\right)}^{2}\right], & \;\text{if}\;\left|t\right|\geq J_{1}, \end{array}\right.$$

where

$$\begin{array}{ccc} & & \Lambda_{1}\left(J_{1},J_{3}\right):=\frac{\vartheta_{1}\left(\vartheta_{3}-\vartheta_{1}\right)J_{1}^{2}}{3{\left(\vartheta_{2}-\vartheta_{1}\right)}^{3}}+\frac{\left(\vartheta_{3}-\vartheta_{1}\right)J_{3}}{\left(\vartheta_{4}-\vartheta_{1}\right)J_{1}},\\ & & \Lambda_{2}\left(J_{1},J_{2}\right):=\frac{\vartheta_{1}\left(\vartheta_{4}-\vartheta_{2}\right)J_{1}^{2}+\vartheta_{1}\left(\vartheta_{4}-\vartheta_{3}-\vartheta_{2}+\vartheta_{1}\right)J_{2}}{\left(\vartheta_{4}-\vartheta_{1}\right)\left(\vartheta_{2}-\vartheta_{1}\right)},\\ & & \Lambda_{3}\left(J_{1},J_{2}\right):=\frac{\vartheta_{1}\left(\vartheta_{3}-\vartheta_{1}\right)J_{1}}{2\left(\vartheta_{2}-\vartheta_{1}\right)}-\frac{\vartheta_{1}J_{1}^{2}+\left(\vartheta_{2}-\vartheta_{1}\right)J_{2}}{J_{1}},\\ & & \Lambda_{4}\left(J_{1},J_{3}\right):=\frac{\vartheta_{1}\left(\vartheta_{3}-\vartheta_{1}\right)J_{1}^{2}}{3{\left(\vartheta_{2}-\vartheta_{1}\right)}^{2}}+\frac{\vartheta_{1}\left(\vartheta_{3}-\vartheta_{1}\right)\left(\vartheta_{2}-\vartheta_{1}\right)J_{3}}{\left(\vartheta_{4}-\vartheta_{1}\right)J_{1}},\\ & & \Lambda_{5}\left(J_{1},J_{2}\right):=\frac{\vartheta_{1}^{2}\left(\vartheta_{4}-\vartheta_{2}\right)J_{1}^{2}+\vartheta_{1}\left(\vartheta_{4}-\vartheta_{3}-\vartheta_{2}+\vartheta_{1}\right)J_{2}}{\vartheta_{4}-\vartheta_{1}}, \end{array}$$

(4.8) $$\rho_{1}≔\frac{\vartheta_{1}J_{1}}{\left(\vartheta_{3}-\vartheta_{1}\right)\left(\vartheta_{2}-\vartheta_{1}\right)}\left(\vartheta_{2}+\vartheta_{3}-\vartheta_{4}-\vartheta_{1}\right)+\frac{2J_{2}}{J_{1}},$$

(4.9) $$\delta_{1}≔\frac{\vartheta_{1}\left(\vartheta_{4}-\vartheta_{1}\right)J_{1}^{2}}{3{\left(\vartheta_{2}-\vartheta_{1}\right)}^{3}}-\frac{\vartheta_{1}^{2}\left(\vartheta_{4}-\vartheta_{2}\right)J_{1}^{2}}{\left(\vartheta_{3}-\vartheta_{1}\right)\left(\vartheta_{2}-\vartheta_{1}\right)}-\frac{\vartheta_{1}\left(\vartheta_{4}-\vartheta_{3}-\vartheta_{2}+\vartheta_{1}\right)J_{2}}{\left(\vartheta_{3}-\vartheta_{1}\right)\left(\vartheta_{2}-\vartheta_{1}\right)}+\frac{J_{3}}{J_{1}},$$

and t is defined by (4.1).

Proof Suppose $u\in S_{q}^{*}\left(r,\Phi \right)$ is of the form (2.8). Then, from (2.11) and (2.12), we have

(4.10) $$\left|\gamma_{2}^{2}-\gamma_{3}^{2}\right|\leq \frac{1}{4r^{2}}\left({\left|a_{r+2}-\frac{1}{2}a_{r+1}^{2}\right|}^{2}+{\left|a_{r+3}-a_{r+1}a_{r+2}+\frac{1}{3}a_{r+1}^{3}\right|}^{2}\right).$$

From (3.4), (3.5) and (3.6) for $u\in S_{q}^{*}\left(r,\Phi \right)$, we obtain

$$\left|a_{r+3}-a_{r+1}a_{r+2}+\frac{1}{3}a_{r+1}^{3}\right|=\frac{\vartheta_{1}J_{1}}{\vartheta_{4}-\vartheta_{1}}\left|L_{3}+\rho_{1}L_{1}L_{2}+\delta_{1}L_{1}^{3}\right|,$$

where $\rho_{1}$ and $\delta_{1}$ are defined by (4.8) and (4.9), respectively. Clearly, $\left(\rho_{1},\delta_{1}\right)$ is a member of either $D_{1},D_{2},D_{3},D_{4}$ or $D_{5}$. Thus, from Lemma 3.2, we get

(4.11) $$\begin{array}{ccc} \left|a_{r+3}-a_{r+1}a_{r+2}+\frac{1}{3}a_{r+1}^{3}\right| & \leq & |\;\frac{\vartheta_{1}^{2}J_{1}^{3}}{3{\left(\vartheta_{2}-\vartheta_{1}\right)}^{3}}+\frac{\vartheta_{1}J_{3}}{\vartheta_{4}-\vartheta_{1}}-\frac{\vartheta_{1}^{2}}{\left(\vartheta_{4}-\vartheta_{1}\right)\left(\vartheta_{3}-\vartheta_{1}\right)\left(\vartheta_{2}-\vartheta_{1}\right)}\\ & & \times \left(\vartheta_{1}\left(\vartheta_{4}-\vartheta_{2}\right)J_{1}^{3}+\left(\vartheta_{4}-\vartheta_{3}-\vartheta_{2}+\vartheta_{1}\right)J_{1}J_{2}\right)|. \end{array}$$

Utilizing the bounds derived from (4.3) and (4.11) in the Equation (4.10), the desired bound is achieved.

Theorem 4.4 *Let*$u\in S_{q}^{C}\left(r,\Phi \right)$*, where*Φ(z)*is given by*(2.7)*. If*$\left(\rho_{2},\delta_{2}\right)\in \cup_{i=1}^{5}D_{i}$*holds, then*

$$\left|T_{2,2}\left(\gamma_{u}\right)\right|\leq \left\{ \begin{array}{cc} \frac{\vartheta_{1}^{4}J_{1}^{2}}{{\left(2r\vartheta_{3}\left(\vartheta_{3}-\vartheta_{1}\right)\right)}^{2}}\left[1+\frac{1}{(\vartheta_{4}^{2}{\left(\vartheta_{4}-\vartheta_{1}\right)}^{2}}{\left(\varphi_{1}\left(J_{1},J_{2}\right)-\varphi_{2}\left(J_{1},J_{2}\right)+\varphi_{3}\left(J_{1},J_{3}\right)\right)}^{2}\right], & \;\text{if}\;\;\left|\varepsilon \right|<J_{1},\\ \frac{\vartheta_{1}^{4}J_{1}^{2}}{{\left(2r\vartheta_{3}\left(\vartheta_{3}-\vartheta_{1}\right)\right)}^{2}}\left[\varphi_{4}^{2}\left(J_{1},J_{2}\right)+\frac{1}{(\vartheta_{4}^{2}{\left(\vartheta_{4}-\vartheta_{1}\right)}^{2}}{\left(\varphi_{1}\left(J_{1},J_{2}\right)-\varphi_{2}\left(J_{1},J_{2}\right)+\varphi_{3}\left(J_{1},J_{3}\right)\right)}^{2}\right], & \;\text{if}\;\;\left|\varepsilon \right|\geq J_{1}, \end{array}\right.$$

where

$$\begin{array}{ccc} & & \varphi_{1}\left(J_{1},J_{2}\right):=\frac{\vartheta_{1}^{4}\vartheta_{3}\left(\vartheta_{4}-\vartheta_{1}\right)\left(\vartheta_{3}-\vartheta_{1}\right)J_{1}^{2}}{3\vartheta_{2}^{2}{\left(\vartheta_{2}-\vartheta_{1}\right)}^{3}}+\frac{\vartheta_{1}\vartheta_{3}\left(\vartheta_{3}+\vartheta_{2}-2\vartheta_{1}\right)J_{2}}{\vartheta_{2}-\vartheta_{1}},\\ & & \varphi_{2}\left(J_{1},J_{2}\right):=\frac{\vartheta_{1}^{2}\vartheta_{4}\left(\vartheta_{4}-\vartheta_{1}\right)\left(\vartheta_{1}J_{1}^{2}+\left(\vartheta_{2}-\vartheta_{1}\right)J_{2}\right)}{\vartheta_{2}{\left(\vartheta_{2}-\vartheta_{1}\right)}^{2}},\\ & & \varphi_{3}\left(J_{1},J_{3}\right):=\frac{\vartheta_{1}\vartheta_{3}J_{1}^{3}+\left(\vartheta_{2}-\vartheta_{1}\right)J_{3}}{\left(\vartheta_{2}-\vartheta_{1}\right)J_{1}},\\ & & \varphi_{4}\left(J_{1},J_{2}\right):=\frac{\vartheta_{1}J_{1}}{\vartheta_{2}-\vartheta_{1}}\left(\frac{\vartheta_{1}\vartheta_{3}\left(\vartheta_{3}-\vartheta_{1}\right)}{2\vartheta_{2}^{2}\left(\vartheta_{2}-\vartheta_{1}\right)}-1\right)-\frac{J_{2}}{J_{1}}, \end{array}$$

(4.12) $$\rho_{2}≔\frac{\vartheta_{1}\left(\vartheta_{3}+\vartheta_{2}-2\vartheta_{1}\right)J_{1}}{\left(\vartheta_{3}-\vartheta_{1}\right)\left(\vartheta_{2}-\vartheta_{1}\right)}-\frac{\vartheta_{1}^{4}\vartheta_{4}\left(\vartheta_{4}-\vartheta_{1}\right)J_{1}}{\vartheta_{3}\vartheta_{2}\left(\vartheta_{3}-\vartheta_{1}\right)\left(\vartheta_{2}-\vartheta_{1}\right)}+\frac{2J_{2}}{J_{1}},$$

(4.13) $$\delta_{2}≔\frac{\vartheta_{1}^{4}\left(\vartheta_{4}-\vartheta_{1}\right)J_{1}^{2}}{3\vartheta_{2}^{2}{\left(\vartheta_{2}-\vartheta_{1}\right)}^{3}}-\frac{\vartheta_{1}^{2}\vartheta_{4}\left(\vartheta_{4}-\vartheta_{2}\right)}{\vartheta_{3}\vartheta_{2}\left(\vartheta_{3}-\vartheta_{1}\right)\left(\vartheta_{2}-\vartheta_{1}\right)}\left(\frac{\vartheta_{1}J_{1}^{2}}{\vartheta_{2}-\vartheta_{1}}+J_{2}\right)+\frac{\vartheta_{1}^{2}J_{1}^{2}}{{\left(\vartheta_{2}-\vartheta_{1}\right)}^{2}}\left(\frac{\vartheta_{3}+\vartheta_{2}-2\vartheta_{1}}{\vartheta_{3}-\vartheta_{1}}-1\right)+\frac{\vartheta_{1}\left(\vartheta_{3}+\vartheta_{2}-2\vartheta_{1}\right)J_{2}}{\left(\vartheta_{3}-\vartheta_{1}\right)\left(\vartheta_{2}-\vartheta_{1}\right)}+\frac{J_{3}}{J_{1}},\;$$

and ε is defined by (4.5).

Proof Suppose $u\in S_{q}^{C}\left(r,\Phi \right)$ is of the form (2.8). Then, from (3.4), (3.5) and (3.6), we obtain

$$\left|a_{r+3}-a_{r+1}a_{r+2}+\frac{1}{3}a_{r+1}^{3}\right|=\frac{\vartheta_{1}^{2}J_{1}}{\vartheta_{4}\left(\vartheta_{4}-\vartheta_{1}\right)}\left|L_{3}+\rho_{2}L_{1}L_{2}+\delta_{2}L_{1}^{3}\right|,$$

where $\rho_{2}$ and $\delta_{2}$ are defined by (4.12) and (4.13) respectively. Clearly, $\left(\rho_{2},\delta_{2}\right)$ is a member of either $D_{1},D_{2},D_{3},D_{4}$ or $D_{5}$. Thus, from Lemma 3.2, we get

(4.14) $$\begin{array}{ccc} \left|a_{r+3}-a_{r+1}a_{r+2}+\frac{1}{3}a_{r+1}^{3}\right|\leq \frac{\vartheta_{1}^{2}J_{1}}{\vartheta_{4}\left(\vartheta_{4}-\vartheta_{1}\right)} & \times & |\frac{\vartheta_{1}^{4}\left(\vartheta_{4}-\vartheta_{1}\right)J_{1}^{2}}{3\vartheta_{2}^{2}{\left(\vartheta_{2}-\vartheta_{1}\right)}^{3}}-\frac{\vartheta_{1}^{2}\vartheta_{4}\left(\vartheta_{4}-\vartheta_{1}\right)\left(\vartheta_{1}J_{1}^{2}+\left(\vartheta_{2}-\vartheta_{1}\right)J_{2}\right)}{\vartheta_{3}\vartheta_{2}\left(\vartheta_{3}-\vartheta_{1}\right){\left(\vartheta_{2}-\vartheta_{1}\right)}^{2}}\\ & & +\frac{\vartheta_{1}^{2}J_{1}^{2}}{\left(\vartheta_{2}-\vartheta_{1}\right)\left(\vartheta_{3}-\vartheta_{1}\right)}+\frac{\vartheta_{1}\left(\vartheta_{3}+\vartheta_{2}-2\vartheta_{1}\right)J_{2}}{\left(\vartheta_{3}-\vartheta_{1}\right)\left(\vartheta_{2}-\vartheta_{1}\right)}+\frac{J_{3}}{J_{1}}|. \end{array}$$

Incorporating the bounds provided by (4.7) and (4.14) into the inequality (4.10) yields the specified bound.

Theorem 4.5 *Let*Φ(z)*be given by*(2.7)*and let*$u\in S_{q}^{*}\left(r,\Phi \right)$*. If*$\left(\rho_{1},\delta_{1}\right)\in \cup_{i=1}^{5}D_{i}$*holds, then*

$$\left|H_{2,1}\left(\gamma_{u}\right)\right|\leq \left\{ \begin{array}{cc} \frac{\vartheta_{1}^{2}J_{1}^{2}}{4r^{2}}\left[\frac{1}{{\left(\vartheta_{3}-\vartheta_{1}\right)}^{2}}+\left|\psi_{1}\left(J_{1},J_{3}\right)-\psi_{2}\left(J_{1},J_{2}\right)\right|\right], & \;\text{if}\;\;\left|t\right|<J_{1},\\ \frac{\vartheta_{1}^{2}J_{1}^{2}}{4r^{2}}\left[\psi_{3}^{2}\left(J_{1},J_{2}\right)+\left|\psi_{1}\left(J_{1},J_{3}\right)-\psi_{2}\left(J_{1},J_{2}\right)\right|\right], & \;\text{if}\;\;\left|t\right|\geq J_{1}, \end{array}\right.$$

where

$$\begin{array}{ccc} & & \psi_{1}\left(J_{1},J_{3}\right):=\frac{\vartheta_{1}J_{1}^{2}}{3{\left(\vartheta_{2}-\vartheta_{1}\right)}^{4}}+\frac{J_{3}}{\left(\vartheta_{4}-\vartheta_{1}\right)\left(\vartheta_{2}-\vartheta_{1}\right)J_{1}},\\ & & \psi_{2}\left(J_{1},J_{2}\right):=\frac{\vartheta_{1}^{2}J_{1}^{2}}{\left(\vartheta_{3}-\vartheta_{1}\right){\left(\vartheta_{2}-\vartheta_{1}\right)}^{2}}+\frac{\vartheta_{1}\left(\vartheta_{4}-\vartheta_{3}-\vartheta_{2}+\vartheta_{1}\right)J_{2}}{\left(\vartheta_{4}-\vartheta_{1}\right)\left(\vartheta_{3}-\vartheta_{1}\right)\left(\vartheta_{2}-\vartheta_{1}\right)},\\ & & \psi_{3}\left(J_{1},J_{2}\right):=\frac{\vartheta_{1}J_{1}}{2{\left(\vartheta_{2}-\vartheta_{1}\right)}^{2}}-\frac{\vartheta_{1}J_{1}}{\left(\vartheta_{3}-\vartheta_{1}\right)\left(\vartheta_{2}-\vartheta_{1}\right)}-\frac{J_{2}}{\left(\vartheta_{3}-\vartheta_{1}\right)J_{1}}, \end{array}$$

$\rho_{1}$ is defined by (4.8), $\delta_{1}$ is defined by (4.9) and t is defined by (4.1).

Proof Suppose $u\in S_{q}^{*}\left(r,\Phi \right)$ is of the form (2.8). Then, from (2.10), (2.11) and (2.12), we have

(4.15) $$\left|\gamma_{1}\gamma_{3}-\gamma_{2}^{2}\right|\leq \frac{1}{4r^{2}}\left({\left|a_{r+2}-\frac{1}{2}a_{r+1}^{2}\right|}^{2}+\left|a_{r+1}\right|\left|a_{r+3}-a_{r+1}a_{r+2}+\frac{1}{3}a_{r+1}^{3}\right|\right).$$

Using the bounds from (4.2), (4.3) and (4.11) in the inequality (4.15), the required bound is obtained.

Theorem 4.6 *Let*$u\in S_{q}^{C}\left(r,\Phi \right)$*, where*Φ(z)*is given by*(2.7)*. If*$\left(\rho_{2},\delta_{2}\right)\in \cup_{i=1}^{5}D_{i}$*holds, then*

$$\left|H_{2,1}\left(\gamma_{u}\right)\right|\leq \left\{ \begin{array}{cc} \frac{\vartheta_{1}^{4}J_{1}^{2}}{4r^{2}}\left[\frac{1}{\vartheta_{3}^{2}{\left(\vartheta_{3}-\vartheta_{1}\right)}^{2}}+\frac{1}{\vartheta_{4}\vartheta_{2}\left(\vartheta_{4}-\vartheta_{1}\right)\left(\vartheta_{2}-\vartheta_{1}\right)}\left|\Xi_{1}\left(J_{1},J_{2}\right)-\Xi_{2}\left(J_{1},J_{2}\right)+\Xi_{3}\left(J_{1},J_{3}\right)\right|\right], & \;\text{if}\;\;\left|\varepsilon \right|<J_{1},\\ \frac{\vartheta_{1}^{4}J_{1}^{2}}{4r^{2}}\left[\frac{\Xi_{4}^{2}\left(J_{1},J_{2}\right)}{\vartheta_{3}^{2}{\left(\vartheta_{3}-\vartheta_{1}\right)}^{2}}+\frac{1}{\vartheta_{4}\vartheta_{2}\left(\vartheta_{4}-\vartheta_{1}\right)\left(\vartheta_{2}-\vartheta_{1}\right)}\left|\Xi_{1}\left(J_{1},J_{2}\right)-\Xi_{2}\left(J_{1},J_{2}\right)+\Xi_{3}\left(J_{1},J_{3}\right)\right|\right], & \;\text{if}\;\left|\varepsilon \right|\geq J_{1}, \end{array}\right.$$

where

$$\begin{array}{ccc} & & \Xi_{1}\left(J_{1},J_{2}\right):=\frac{\vartheta_{1}^{4}\left(\vartheta_{4}-\vartheta_{1}\right)J_{1}^{2}}{3\vartheta_{2}^{2}{\left(\vartheta_{2}-\vartheta_{1}\right)}^{3}}+\frac{\vartheta_{1}\left(\vartheta_{3}+\vartheta_{2}-2\vartheta_{1}\right)J_{2}}{\left(\vartheta_{3}-\vartheta_{1}\right)\left(\vartheta_{2}-\vartheta_{1}\right)},\\ & & \Xi_{2}\left(J_{1},J_{2}\right):=\frac{\vartheta_{1}^{2}\vartheta_{4}\left(\vartheta_{4}-\vartheta_{1}\right)\left(\vartheta_{1}J_{1}^{2}+\left(\vartheta_{2}-\vartheta_{1}\right)J_{2}\right)}{\vartheta_{3}\vartheta_{2}\left(\vartheta_{3}-\vartheta_{1}\right){\left(\vartheta_{2}-\vartheta_{1}\right)}^{2}},\\ & & \Xi_{3}\left(J_{1},J_{3}\right):=\frac{\vartheta_{1}^{2}J_{1}^{2}}{\left(\vartheta_{2}-\vartheta_{1}\right)\left(\vartheta_{3}-\vartheta_{1}\right)}+\frac{J_{3}}{J_{1}},\\ & & \Xi_{4}\left(J_{1},J_{2}\right):=\frac{\vartheta_{1}^{2}\vartheta_{3}\left(\vartheta_{3}-\vartheta_{1}\right)J_{1}}{2\vartheta_{2}^{2}{\left(\vartheta_{2}-\vartheta_{1}\right)}^{2}}-\frac{\vartheta_{1}J_{1}^{2}+\left(\vartheta_{2}-\vartheta_{1}\right)J_{2}}{\left(\vartheta_{2}-\vartheta_{1}\right)J_{1}}, \end{array}$$

$\rho_{2}$ is defined by (4.12), $\delta_{2}$ is defined by (4.13) and ε is defined by (4.5).

Proof By applying the bounds established in (4.6), (4.7) and (4.14) to the inequality (4.15), the necessary bound is achieved.

**Consequences for the family** $S_{q}^{*}\left(r,\Phi \right)$

In this section, we delve into the analysis of some Ma-Minda type functions, Φ(z), presenting several corollaries that arise under the scenario where $J_{1}>0$ and $J_{2}$ with $J_{3}$ being any real number.

If we take Φ(z) as defined in (2.17), then Theorems 4.1, 4.3 and 4.5 guide us to the ensuing corollaries:

Corollary 5.1 *Let*$u\in S_{q}^{*}\left(r,A,B\right)$*be of the form*(2.8)*. Then*

$$\left|T_{2,1}\left(\gamma_{u}\right)\right|\leq \left\{ \begin{array}{cc} \frac{\vartheta_{1}^{2}{\left(A-B\right)}^{2}}{4r^{2}}\left[\frac{1}{{\left(\vartheta_{2}-\vartheta_{1}\right)}^{2}}+\frac{1}{\vartheta_{3}-\vartheta_{1}}\right], & \;\text{if}\;\left|s\right|<1-A,\\ \frac{\vartheta_{1}^{2}{\left(A-B\right)}^{2}}{4r^{2}{\left(\vartheta_{2}-\vartheta_{1}\right)}^{2}}\left[1+\vartheta_{1}^{2}{\left(\frac{A-B}{2\left(\vartheta_{2}-\vartheta_{1}\right)}-\frac{A^{2}-\left(\vartheta_{2}-\vartheta_{1}+2\right)\mathrm{AB}+\left(\vartheta_{2}-\vartheta_{1}+1\right)B^{2}}{\left(\vartheta_{3}-\vartheta_{1}\right)\left(A-B\right)}\right)}^{2}\right], & \;\text{if}\;\left|s\right|\geq 1-A, \end{array}\right.$$

where

(5.1) $$s≔\frac{\left(\vartheta_{1}\left(\vartheta_{3}-\vartheta_{1}\right)-2\vartheta_{2}\left(\vartheta_{2}-\vartheta_{1}\right)\right)\left(A-B\right)}{2{\left(\vartheta_{2}-\vartheta_{1}\right)}^{2}}.$$

Corollary 5.2 *Let*$u\in S_{q}^{*}\left(r,A,B\right)$*be of the form*(2.8)*and*$\left(\rho_{3},\delta_{3}\right)\in \cup_{i=1}^{5}D_{i}$*holds. Then*

$$\left|T_{2,2}\left(\gamma_{u}\right)\right|\leq \left\{ \begin{array}{cc} \frac{\vartheta_{1}^{2}{\left(A-B\right)}^{2}}{4r^{2}{\left(\vartheta_{3}-\vartheta_{1}\right)}^{2}}\left[1+{\left(\Lambda_{1}\left(A,B\right)-\Lambda_{2}\left(A,B\right)\right)}^{2}\right], & \;\text{if}\;\left|s\right|<1-A,\\ \frac{\vartheta_{1}^{2}{\left(A-B\right)}^{2}}{4r^{2}{\left(\vartheta_{3}-\vartheta_{1}\right)}^{2}{\left(\vartheta_{2}-\vartheta_{1}\right)}^{2}}\left[\Lambda_{3}^{2}\left(A,B\right)+{\left(\Lambda_{4}\left(A,B\right)-\Lambda_{5}\left(A,B\right)\right)}^{2}\right], & \;\text{if}\;\left|s\right|\geq 1-A, \end{array}\right.$$

where

$$\begin{array}{ccc} & & \Lambda_{1}\left(A,B\right):=\frac{\vartheta_{1}\left(\vartheta_{3}-\vartheta_{1}\right){\left(A-B\right)}^{2}}{3{\left(\vartheta_{2}-\vartheta_{1}\right)}^{3}}+\frac{\left(\vartheta_{3}-\vartheta_{1}\right)B^{2}}{\vartheta_{4}-\vartheta_{1}},\\ & & \Lambda_{2}\left(A,B\right):=\frac{\vartheta_{1}\left(\left(\vartheta_{4}-\vartheta_{2}\right)\left(A-2B\right)+\left(\vartheta_{3}-\vartheta_{1}\right)B\right)\left(A-B\right)}{\left(\vartheta_{4}-\vartheta_{1}\right)\left(\vartheta_{2}-\vartheta_{1}\right)},\\ & & \Lambda_{3}\left(A,B\right):=\frac{\vartheta_{1}\left(\vartheta_{3}-\vartheta_{1}\right)\left(A-B\right)}{2\left(\vartheta_{2}-\vartheta_{1}\right)}+\vartheta_{2}B-\vartheta_{1}A,\\ & & \Lambda_{4}\left(A,B\right):=\frac{\vartheta_{1}\left(\vartheta_{3}-\vartheta_{1}\right){\left(A-B\right)}^{2}}{3{\left(\vartheta_{2}-\vartheta_{1}\right)}^{2}}+\frac{\vartheta_{1}\left(\vartheta_{3}-\vartheta_{1}\right)\left(\vartheta_{2}-\vartheta_{1}\right)B^{2}}{\vartheta_{4}-\vartheta_{1}},\\ & & \Lambda_{5}\left(A,B\right):=\frac{\vartheta_{1}\left(\vartheta_{1}\left(\vartheta_{4}-\vartheta_{2}\right)\left(A-B\right)-\left(\vartheta_{4}-\vartheta_{3}-\vartheta_{2}+\vartheta_{1}\right)B\right)\left(A-B\right)}{\vartheta_{4}-\vartheta_{1}}, \end{array}$$

(5.2) $$\rho_{3}≔\frac{\vartheta_{1}\left(\vartheta_{2}+\vartheta_{3}-\vartheta_{4}-\vartheta_{1}\right)\left(A-B\right)}{\left(\vartheta_{3}-\vartheta_{1}\right)\left(\vartheta_{2}-\vartheta_{1}\right)}-B,$$

(5.3) $$\delta_{3}≔\frac{\vartheta_{1}\left(\vartheta_{4}-\vartheta_{1}\right){\left(A-B\right)}^{2}}{3{\left(\vartheta_{2}-\vartheta_{1}\right)}^{3}}-\frac{\vartheta_{1}^{2}\left(\vartheta_{4}-\vartheta_{2}\right){\left(A-B\right)}^{2}+\vartheta_{1}\left(\vartheta_{4}-\vartheta_{3}-\vartheta_{2}+\vartheta_{1}\right)\left(B^{2}-AB\right)}{\left(\vartheta_{3}-\vartheta_{1}\right)\left(\vartheta_{2}-\vartheta_{1}\right)}+B^{2},$$

and s is defined by (5.1).

Corollary 5.3 *Let*$u\in S_{q}^{*}\left(r,A,B\right)$*be of the form*(2.8)*and*$\left(\rho_{3},\delta_{3}\right)\in \cup_{i=1}^{5}D_{i}$*holds. Then*

$$\left|H_{2,1}\left(\gamma_{u}\right)\right|\leq \left\{ \begin{array}{cc} \frac{\vartheta_{1}^{2}{\left(A-B\right)}^{2}}{4r^{2}}\left[\frac{1}{{\left(\vartheta_{3}-\vartheta_{1}\right)}^{2}}+\left|\psi_{1}\left(A,B\right)-\psi_{2}\left(A,B\right)\right|\right], & \;\text{if}\;\left|s\right|<1-A,\\ \frac{\vartheta_{1}^{2}{\left(A-B\right)}^{2}}{4r^{2}}\left[\psi_{3}^{2}\left(A,B\right)+\left|\psi_{1}\left(A,B\right)-\psi_{2}\left(A,B\right)\right|\right], & \;\text{if}\;\left|s\right|\geq 1-A, \end{array}\right.$$

where

$$\begin{array}{ccc} & & \psi_{1}\left(A,B\right):=\frac{\vartheta_{1}{\left(A-B\right)}^{2}}{3{\left(\vartheta_{2}-\vartheta_{1}\right)}^{4}}+\frac{B^{2}}{\left(\vartheta_{4}-\vartheta_{1}\right)\left(\vartheta_{2}-\vartheta_{1}\right)},\\ & & \psi_{2}\left(A,B\right):=\frac{\vartheta_{1}^{2}{\left(A-B\right)}^{2}}{\left(\vartheta_{3}-\vartheta_{1}\right){\left(\vartheta_{2}-\vartheta_{1}\right)}^{2}}-\frac{\vartheta_{1}\left(\vartheta_{4}-\vartheta_{3}-\vartheta_{2}+\vartheta_{1}\right)B\left(A-B\right)}{\left(\vartheta_{4}-\vartheta_{1}\right)\left(\vartheta_{3}-\vartheta_{1}\right)\left(\vartheta_{2}-\vartheta_{1}\right)}\\ & & \psi_{3}\left(A,B\right):=\frac{\vartheta_{1}\left(A-B\right)}{2{\left(\vartheta_{2}-\vartheta_{1}\right)}^{2}}+\frac{\vartheta_{2}B-\vartheta_{1}A}{\left(\vartheta_{3}-\vartheta_{1}\right)\left(\vartheta_{2}-\vartheta_{1}\right)}, \end{array}$$

$\rho_{3}$ is defined by (5.2), $\delta_{3}$ is defined by (5.3) and s is defined by (5.1). If we set Φ(z) as defined in (2.20), then Theorems 4.1, 4.3 and 4.5 guide us to the subsequent corollaries:

Corollary 5.4 *Let*$u\in S_{q}^{*}\left(r,\alpha \right)$*be of the form*(2.8)*. Then*

$$\left|T_{2,1}\left(\gamma_{u}\right)\right|\leq \left\{ \begin{array}{cc} \frac{\vartheta_{1}^{2}{\left(1-\alpha \right)}^{2}}{r^{2}}\left[\frac{1}{{\left(\vartheta_{2}-\vartheta_{1}\right)}^{2}}+\frac{1}{\vartheta_{3}-\vartheta_{1}}\right], & \;\text{if}\;\left|u\right|<\alpha ,\\ \frac{\vartheta_{1}^{2}{\left(1-\alpha \right)}^{2}}{r^{2}{\left(\vartheta_{2}-\vartheta_{1}\right)}^{2}}\left[1+\vartheta_{1}^{2}{\left(\frac{1-\alpha }{\vartheta_{2}-\vartheta_{1}}-\frac{\vartheta_{2}-\vartheta_{1}+2\left(1-\alpha \right)}{\vartheta_{3}-\vartheta_{1}}\right)}^{2}\right], & \;\text{if}\;\;\left|u\right|\geq \alpha , \end{array}\right.$$

where

(5.4) $$u≔\frac{\left(\vartheta_{1}\left(\vartheta_{3}-\vartheta_{1}\right)-2\vartheta_{2}\left(\vartheta_{2}-\vartheta_{1}\right)\right)\left(1-\alpha \right)}{2{\left(\vartheta_{2}-\vartheta_{1}\right)}^{2}}.$$

Corollary 5.5 *Let*$u\in S_{q}^{*}\left(r,\alpha \right)$*be of the form*(2.8)*and*$\left(\rho_{4},\delta_{4}\right)\in \cup_{i=1}^{5}D_{i}$*holds. Then*

$$\left|T_{2,2}\left(\gamma_{u}\right)\right|\leq \left\{ \begin{array}{cc} \frac{\vartheta_{1}^{2}{\left(1-\alpha \right)}^{2}}{r^{2}{\left(\vartheta_{3}-\vartheta_{1}\right)}^{2}}\left[1+{\left(\Lambda_{1}\left(\alpha \right)-\Lambda_{2}\left(\alpha \right)\right)}^{2}\right], & \;\text{if}\;\left|u\right|<\alpha ,\\ \frac{\vartheta_{1}^{2}{\left(1-\alpha \right)}^{2}}{r^{2}{\left(\vartheta_{3}-\vartheta_{1}\right)}^{2}{\left(\vartheta_{2}-\vartheta_{1}\right)}^{2}}\left[\Lambda_{3}^{2}\left(\alpha \right)+{\left(\Lambda_{4}\left(\alpha \right)-\Lambda_{5}\left(\alpha \right)\right)}^{2}\right], & \;\text{if}\;\left|u\right|\geq \alpha , \end{array}\right.$$

where

$$\begin{array}{ccc} & & \Lambda_{1}\left(\alpha \right):=\frac{4\vartheta_{1}\left(\vartheta_{3}-\vartheta_{1}\right){\left(1-\alpha \right)}^{2}}{3{\left(\vartheta_{2}-\vartheta_{1}\right)}^{3}}+\frac{\left(\vartheta_{3}-\vartheta_{1}\right)}{\vartheta_{4}-\vartheta_{1}},\\ & & \Lambda_{2}\left(\alpha \right):=\frac{2\vartheta_{1}\left(-2\left(\vartheta_{4}-\vartheta_{2}\right)\alpha +3\vartheta_{4}-\vartheta_{3}-3\vartheta_{2}+\vartheta_{1}\right)\left(1-\alpha \right)}{\left(\vartheta_{4}-\vartheta_{1}\right)\left(\vartheta_{2}-\vartheta_{1}\right)},\\ & & \Lambda_{3}\left(\alpha \right):=\frac{\vartheta_{1}\left(\vartheta_{3}-\vartheta_{1}\right)\left(1-\alpha \right)-{\left(\vartheta_{2}+\vartheta_{1}\right)}^{2}}{\vartheta_{2}-\vartheta_{1}}+2\vartheta_{1}\alpha ,\\ & & \Lambda_{4}\left(\alpha \right):=\frac{4\vartheta_{1}\left(\vartheta_{3}-\vartheta_{1}\right){\left(1-\alpha \right)}^{2}}{3{\left(\vartheta_{2}-\vartheta_{1}\right)}^{2}}+\frac{\vartheta_{1}\left(\vartheta_{3}-\vartheta_{1}\right)\left(\vartheta_{2}-\vartheta_{1}\right)}{\vartheta_{4}-\vartheta_{1}},\\ & & \Lambda_{5}\left(\alpha \right):=\frac{2\vartheta_{1}\left(2\vartheta_{1}\left(\vartheta_{4}-\vartheta_{2}\right)\left(1-\alpha \right)+\vartheta_{4}-\vartheta_{3}-\vartheta_{2}+\vartheta_{1}\right)\left(1-\alpha \right)}{\vartheta_{4}-\vartheta_{1}}, \end{array}$$

(5.5) $$\rho_{4}≔\frac{2\vartheta_{1}\left(\vartheta_{2}+\vartheta_{3}-\vartheta_{4}-\vartheta_{1}\right)\left(1-\alpha \right)}{\left(\vartheta_{3}-\vartheta_{1}\right)\left(\vartheta_{2}-\vartheta_{1}\right)}+1,\;$$

(5.6) $$\delta_{4}≔\frac{4\vartheta_{1}\left(\vartheta_{4}-\vartheta_{1}\right){\left(1-\alpha \right)}^{2}}{3{\left(\vartheta_{2}-\vartheta_{1}\right)}^{3}}-\frac{4\vartheta_{1}^{2}\left(\vartheta_{4}-\vartheta_{2}\right){\left(1-\alpha \right)}^{2}+2\vartheta_{1}\left(\vartheta_{4}-\vartheta_{3}-\vartheta_{2}+\vartheta_{1}\right)\left(1-\alpha \right)}{\left(\vartheta_{3}-\vartheta_{1}\right)\left(\vartheta_{2}-\vartheta_{1}\right)}+1,$$

and u is defined by (5.4).

Corollary 5.6 *Let*$u\in S_{q}^{*}\left(r,\alpha \right)$*be of the form*(2.8)*and*$\left(\rho_{4},\delta_{4}\right)\in \cup_{i=1}^{5}D_{i}$*holds. Then*

$$\left|H_{2,1}\left(\gamma_{u}\right)\right|\leq \left\{ \begin{array}{cc} \frac{\vartheta_{1}^{2}{\left(1-\alpha \right)}^{2}}{r^{2}}\left[\frac{1}{{\left(\vartheta_{3}-\vartheta_{1}\right)}^{2}}+\left|\psi_{1}\left(\alpha \right)+\psi_{2}\left(\alpha \right)\right|\right],\; & \;\text{if}\;\;\left|u\right|<\alpha ,\\ \frac{\vartheta_{1}^{2}{\left(1-\alpha \right)}^{2}}{r^{2}}\left[\psi_{3}^{2}\left(\alpha \right)+\left|\psi_{1}\left(\alpha \right)+\psi_{2}\left(\alpha \right)\right|\right], & \;\text{if}\;\;\left|u\right|\geq \alpha , \end{array}\right.$$

where

$$\begin{array}{ccc} & & \psi_{1}\left(\alpha \right):=\frac{\vartheta_{3}-\vartheta_{1}-2\vartheta_{1}\left(\vartheta_{4}-\vartheta_{3}-\vartheta_{2}+\vartheta_{1}\right)\left(1-\alpha \right)}{\left(\vartheta_{4}-\vartheta_{1}\right)\left(\vartheta_{3}-\vartheta_{1}\right)\left(\vartheta_{2}-\vartheta_{1}\right)},\\ & & \psi_{2}\left(\alpha \right):=\frac{4\vartheta_{1}{\left(1-\alpha \right)}^{2}}{3{\left(\vartheta_{2}-\vartheta_{1}\right)}^{4}}-\frac{4\vartheta_{1}^{2}{\left(1-\alpha \right)}^{2}}{\left(\vartheta_{3}-\vartheta_{1}\right){\left(\vartheta_{2}-\vartheta_{1}\right)}^{2}},\\ & & \psi_{3}\left(\alpha \right):=\frac{\vartheta_{1}\left(1-\alpha \right)}{{\left(\vartheta_{2}-\vartheta_{1}\right)}^{2}}-\frac{\vartheta_{2}+\vartheta_{1}\left(1-2\alpha \right)}{\left(\vartheta_{3}-\vartheta_{1}\right)\left(\vartheta_{2}-\vartheta_{1}\right)}, \end{array}$$

$\rho_{4}$ is defined by (5.5), $\delta_{4}$ is defined by (5.6) and u is defined by (5.4). If we define Φ(z) as given in (2.23), then Theorems 4.1, 4.3 and 4.5 guide us to the ensuing corollaries:

Corollary 5.7 *Let*$u\in S_{q}^{*}\left(r,\beta \right)$*be of the form*(2.8)*. Then*

$$\left|T_{2,1}\left(\gamma_{u}\right)\right|\leq \left\{ \begin{array}{cc} \frac{\vartheta_{1}^{2}\beta^{2}}{r^{2}}\left[\frac{1}{{\left(\vartheta_{2}-\vartheta_{1}\right)}^{2}}+\frac{1}{\vartheta_{3}-\vartheta_{1}}\right], & \;\text{if}\;\left|v\right|<1-\beta ,\\ \frac{\vartheta_{1}^{4}\beta^{4}}{r^{2}{\left(\vartheta_{2}-\vartheta_{1}\right)}^{2}}\left[\frac{1}{\vartheta_{1}^{2}\beta^{2}}+{\left(\frac{1}{\vartheta_{2}-\vartheta_{1}}-\frac{\vartheta_{2}-\vartheta_{1}+2}{\vartheta_{3}-\vartheta_{1}}\right)}^{2}\right], & \;\text{if}\;\left|v\right|\geq 1-\beta , \end{array}\right.$$

where

(5.7) $$v≔\frac{\left(\vartheta_{1}\left(\vartheta_{3}-\vartheta_{1}\right)-2\vartheta_{2}\left(\vartheta_{2}-\vartheta_{1}\right)\right)\beta }{{\left(\vartheta_{2}-\vartheta_{1}\right)}^{2}}.$$

Corollary 5.8 *Let*$u\in S_{q}^{*}\left(r,\beta \right)$*be of the form*(2.8)*and*$\left(\rho_{5},\delta_{5}\right)\in \cup_{i=1}^{5}D_{i}$*holds. Then*

$$\left|T_{2,2}\left(\gamma_{u}\right)\right|\leq \left\{ \begin{array}{cc} \frac{\vartheta_{1}^{2}\beta^{2}}{r^{2}{\left(\vartheta_{3}-\vartheta_{1}\right)}^{2}}\left[1+{\left(\Lambda_{1}\left(\beta \right)-\Lambda_{2}\left(\beta \right)\right)}^{2}\right], & \;\text{if}\;\left|v\right|<1-\beta ,\\ \frac{\vartheta_{1}^{2}\beta^{2}}{r^{2}{\left(\vartheta_{3}-\vartheta_{1}\right)}^{2}{\left(\vartheta_{2}-\vartheta_{1}\right)}^{2}}\left[\Lambda_{3}^{2}\left(\beta \right)+{\left(\Lambda_{4}\left(\beta \right)-\Lambda_{5}\left(\beta \right)\right)}^{2}\right], & \;\text{if}\;\left|v\right|\geq 1-\beta , \end{array}\right.$$

where

$$\begin{array}{ccc} & & \Lambda_{1}\left(\beta \right):=\frac{4\vartheta_{1}\left(\vartheta_{3}-\vartheta_{1}\right)\beta^{2}}{3{\left(\vartheta_{2}-\vartheta_{1}\right)}^{3}}+\frac{\left(\vartheta_{3}-\vartheta_{1}\right)\beta^{2}}{\vartheta_{4}-\vartheta_{1}},\\ & & \Lambda_{2}\left(\beta \right):=\frac{2\vartheta_{1}\left(3\vartheta_{4}-\vartheta_{3}-3\vartheta_{2}+\vartheta_{1}\right)\beta^{2}}{\left(\vartheta_{4}-\vartheta_{1}\right)\left(\vartheta_{2}-\vartheta_{1}\right)},\\ & & \Lambda_{3}\left(\beta \right):=\frac{\left(\vartheta_{1}\vartheta_{3}-\vartheta_{2}^{2}\right)\beta }{\vartheta_{2}-\vartheta_{1}},\\ & & \Lambda_{4}\left(\beta \right):=\frac{4\vartheta_{1}\left(\vartheta_{3}-\vartheta_{1}\right)\beta^{2}}{3{\left(\vartheta_{2}-\vartheta_{1}\right)}^{2}}+\frac{\vartheta_{1}\left(\vartheta_{3}-\vartheta_{1}\right)\left(\vartheta_{2}-\vartheta_{1}\right)\beta^{2}}{\vartheta_{4}-\vartheta_{1}},\\ & & \Lambda_{5}\left(\beta \right):=\frac{2\vartheta_{1}\left(\left(2\vartheta_{1}+1\right)\left(\vartheta_{4}-\vartheta_{2}\right)-\left(\vartheta_{3}-\vartheta_{1}\right)\right)\beta^{2}}{\vartheta_{4}-\vartheta_{1}}, \end{array}$$

(5.8) $$\rho_{5}≔\frac{2\vartheta_{1}\left(\vartheta_{2}+\vartheta_{3}-\vartheta_{4}-\vartheta_{1}\right)\beta }{\left(\vartheta_{3}-\vartheta_{1}\right)\left(\vartheta_{2}-\vartheta_{1}\right)}+\beta ,$$

(5.9) $$\delta_{5}≔\frac{4\vartheta_{1}\left(\vartheta_{4}-\vartheta_{1}\right)\beta^{2}}{3{\left(\vartheta_{2}-\vartheta_{1}\right)}^{3}}-\frac{4\vartheta_{1}^{2}\left(\vartheta_{4}-\vartheta_{2}\right)\beta^{2}+2\vartheta_{1}\left(\vartheta_{4}-\vartheta_{3}-\vartheta_{2}+\vartheta_{1}\right)\beta^{2}}{\left(\vartheta_{3}-\vartheta_{1}\right)\left(\vartheta_{2}-\vartheta_{1}\right)}+\beta^{2},$$

and v is defined by (5.7).

Corollary 5.9 *Let*$u\in S_{q}^{*}\left(r,\beta \right)$*be of the form*(2.8)*and*$\left(\rho_{5},\delta_{5}\right)\in \cup_{i=1}^{5}D_{i}$*holds. Then*

$$\left|H_{2,1}\left(\gamma_{u}\right)\right|\leq \left\{ \begin{array}{cc} \frac{\vartheta_{1}^{2}\beta^{2}}{r^{2}}\left[\frac{1}{{\left(\vartheta_{3}-\vartheta_{1}\right)}^{2}}+\left|\psi_{1}\left(\beta \right)-\psi_{2}\left(\beta \right)\right|\right], & \;\text{if}\;\left|v\right|<1-\beta ,\\ \frac{\vartheta_{1}^{2}\beta^{2}}{r^{2}}\left[\psi_{3}^{2}\left(\beta \right)+\left|\psi_{1}\left(\beta \right)-\psi_{2}\left(\beta \right)\right|\right], & \;\text{if}\;\left|v\right|\geq 1-\beta , \end{array}\right.$$

where

$$\begin{array}{ccc} & & \psi_{1}\left(\beta \right):=\frac{4\vartheta_{1}\beta^{2}}{3{\left(\vartheta_{2}-\vartheta_{1}\right)}^{4}}+\frac{\beta^{2}}{\left(\vartheta_{4}-\vartheta_{1}\right)\left(\vartheta_{2}-\vartheta_{1}\right)},\\ & & \psi_{2}\left(\beta \right):=\frac{4\vartheta_{1}^{2}\beta^{2}}{\left(\vartheta_{3}-\vartheta_{1}\right){\left(\vartheta_{2}-\vartheta_{1}\right)}^{2}}+\frac{2\vartheta_{1}\left(\vartheta_{4}-\vartheta_{3}-\vartheta_{2}+\vartheta_{1}\right)\beta^{2}}{\left(\vartheta_{4}-\vartheta_{1}\right)\left(\vartheta_{3}-\vartheta_{1}\right)\left(\vartheta_{2}-\vartheta_{1}\right)},\\ & & \psi_{3}\left(\beta \right):=\frac{(\vartheta_{1}\left(\vartheta_{3}-\vartheta_{1}\right)-{\left(\vartheta_{2}-\vartheta_{1}\right)}^{2})\beta }{\left(\vartheta_{3}-\vartheta_{1}\right){\left(\vartheta_{2}-\vartheta_{1}\right)}^{2}}, \end{array}$$

$\rho_{5}$ is defined by (5.8), $\delta_{5}$ is defined by (5.9) and v is defined by (5.7).

**Consequences for the family** $S_{q}^{C}\left(r,\Phi \right)$

If we take Φ(z) as defined in (2.17), then Theorems 4.2, 4.4 and 4.6 direct us to the ensuing corollaries:

Corollary 6.1 *Let*$u\in S_{q}^{C}\left(r,A,B\right)$*be of the form*(2.8)*. Then*

$$\left|T_{2,1}\left(\gamma_{u}\right)\right|\leq \left\{ \begin{array}{cc} \frac{\vartheta_{1}^{4}{\left(A-B\right)}^{2}}{4r^{2}}\left[\frac{1}{\vartheta_{3}^{2}{\left(\vartheta_{3}-\vartheta_{1}\right)}^{2}}+\frac{1}{\vartheta_{2}^{2}{\left(\vartheta_{2}-\vartheta_{1}\right)}^{2}}\right], & \;\text{if}\;\left|k\right|<1-A,\\ \frac{\vartheta_{1}^{4}{\left(A-B\right)}^{2}}{4r^{2}\vartheta_{3}^{2}{\left(\vartheta_{3}-\vartheta_{1}\right)}^{2}}\left[1+{\left(\frac{\vartheta^{2}\vartheta_{3}\left(\vartheta_{3}-\vartheta_{1}\right)\left(A-B\right)}{2\vartheta_{2}^{2}{\left(\vartheta_{2}-\vartheta_{1}\right)}^{2}}+\frac{\vartheta_{2}B-\vartheta_{1}A}{\vartheta_{2}-\vartheta_{1}}\right)}^{2}\right], & \;\text{if}\;\left|k\right|\geq 1-A, \end{array}\right.$$

where

(6.1) $$k≔\left(\frac{\vartheta_{1}^{2}\vartheta_{3}\left(\vartheta_{3}-\vartheta_{1}\right)}{2\vartheta_{2}^{2}{\left(\vartheta_{2}-\vartheta_{1}\right)}^{2}}-\frac{\vartheta_{2}}{\vartheta_{2}-\vartheta_{1}}\right)\left(A-B\right).$$

Corollary 6.2 *Let*$u\in S_{q}^{C}\left(r,A,B\right)$*be of the form*(2.8)*and*$\left(\rho_{6},\delta_{6}\right)\in \cup_{i=1}^{5}D_{i}$*holds. Then*

$$\left|T_{2,2}\left(\gamma_{u}\right)\right|\leq \left\{ \begin{array}{cc} \frac{\vartheta_{1}^{4}{\left(A-B\right)}^{2}}{4r^{2}\vartheta_{3}^{2}{\left(\vartheta_{3}-\vartheta_{1}\right)}^{2}}\left[1+\frac{1}{\vartheta_{4}^{2}{\left(\vartheta_{4}-\vartheta_{1}\right)}^{2}}{\left(\varphi_{1}\left(A,B\right)+\varphi_{2}\left(A,B\right)+\varphi_{3}\left(A,B\right)\right)}^{2}\right], & \;\text{if}\;\left|k\right|<1-A,\\ \frac{\vartheta_{1}^{4}{\left(A-B\right)}^{2}}{4r^{2}\vartheta_{3}^{2}{\left(\vartheta_{3}-\vartheta_{1}\right)}^{2}}\left[\varphi_{4}^{2}\left(A,B\right)+\frac{1}{\vartheta_{4}^{2}{\left(\vartheta_{4}-\vartheta_{1}\right)}^{2}}{\left(\varphi_{1}\left(A,B\right)+\varphi_{2}\left(A,B\right)+\varphi_{3}\left(A,B\right)\right)}^{2}\right], & \;\text{if}\;\left|k\right|\geq 1-A, \end{array}\right.$$

where

$$\begin{array}{ccc} & & \varphi_{1}\left(A,B\right):=\frac{\vartheta_{1}^{4}\vartheta_{3}\left(\vartheta_{4}-\vartheta_{1}\right)\left(\vartheta_{3}-\vartheta_{1}\right){\left(A-B\right)}^{2}}{3\vartheta_{2}^{2}{\left(\vartheta_{2}-\vartheta_{1}\right)}^{3}}-\frac{\vartheta_{1}\vartheta_{3}\left(\vartheta_{3}+\vartheta_{2}-2\vartheta_{1}\right)B\left(A-B\right)}{\vartheta_{2}-\vartheta_{1}},\\ & & \varphi_{2}\left(A,B\right):=\frac{\vartheta_{1}^{2}\vartheta_{4}\left(\vartheta_{4}-\vartheta_{1}\right)\left(\vartheta_{2}B-\vartheta_{1}A\right)\left(A-B\right)}{\vartheta_{2}{\left(\vartheta_{2}-\vartheta_{1}\right)}^{2}},\\ & & \varphi_{3}\left(A,B\right):=\frac{\vartheta_{1}\vartheta_{3}{\left(A-B\right)}^{2}+\left(\vartheta_{2}-\vartheta_{1}\right)B^{2}}{\vartheta_{2}-\vartheta_{1}},\\ & & \varphi_{4}\left(A,B\right):=\frac{\vartheta_{1}^{2}\vartheta_{3}\left(\vartheta_{3}-\vartheta_{1}\right)\left(A-B\right)}{2\vartheta_{2}^{2}{\left(\vartheta_{2}-\vartheta_{1}\right)}^{2}}+\frac{\vartheta_{2}B-\vartheta_{1}A}{\vartheta_{2}-\vartheta_{1}}, \end{array}$$

(6.2) $$\rho_{6}≔\frac{\vartheta_{1}\left(\vartheta_{3}+\vartheta_{2}-2\vartheta_{1}\right)\left(A-B\right)}{\left(\vartheta_{3}-\vartheta_{1}\right)\left(\vartheta_{2}-\vartheta_{1}\right)}-\frac{\vartheta_{1}^{4}\vartheta_{4}\left(\vartheta_{4}-\vartheta_{1}\right)\left(A-B\right)}{\vartheta_{3}\vartheta_{2}\left(\vartheta_{3}-\vartheta_{1}\right)\left(\vartheta_{2}-\vartheta_{1}\right)}-2B,$$

(6.3) $$\begin{array}{ccc} \delta_{6} & ≔ & \frac{\vartheta_{1}^{4}\left(\vartheta_{4}-\vartheta_{1}\right){\left(A-B\right)}^{2}}{3\vartheta_{2}^{2}{\left(\vartheta_{2}-\vartheta_{1}\right)}^{3}}-\frac{\vartheta_{1}^{2}\vartheta_{4}\left(\vartheta_{4}-\vartheta_{2}\right)}{\vartheta_{3}\vartheta_{2}\left(\vartheta_{3}-\vartheta_{1}\right)\left(\vartheta_{2}-\vartheta_{1}\right)}\left(\frac{\vartheta_{1}{\left(A-B\right)}^{2}}{\vartheta_{2}-\vartheta_{1}}+B^{2}-AB\right)+\frac{\vartheta_{1}^{2}{\left(A-B\right)}^{2}}{{\left(\vartheta_{2}-\vartheta_{1}\right)}^{2}}\left(\frac{\vartheta_{3}+\vartheta_{2}-2\vartheta_{1}}{\vartheta_{3}-\vartheta_{1}}-1\right)\\ & & +\;\frac{\vartheta_{1}\left(\vartheta_{3}+\vartheta_{2}-2\vartheta_{1}\right)B\left(A-B\right)}{\left(\vartheta_{3}-\vartheta_{1}\right)\left(\vartheta_{2}-\vartheta_{1}\right)}+B^{2}, \end{array}$$

and k is defined by (6.1).

Corollary 6.3 *Let*$u\in S_{q}^{C}\left(r,A,B\right)$*be of the form*(2.8)*and*$\left(\rho_{6},\delta_{6}\right)\in \cup_{i=1}^{5}D_{i}$*holds. Then*

$$\left|H_{2,1}\left(\gamma_{u}\right)\right|\leq \left\{ \begin{array}{cc} \frac{\vartheta_{1}^{4}{\left(A-B\right)}^{2}}{4r^{2}}\left[\frac{1}{\vartheta_{3}^{2}{\left(\vartheta_{3}-\vartheta_{1}\right)}^{2}}+\frac{1}{\vartheta_{4}\vartheta_{2}\left(\vartheta_{4}-\vartheta_{1}\right)\left(\vartheta_{2}-\vartheta_{1}\right)}\left|\Xi_{1}\left(A,B\right)+\Xi_{2}\left(A,B\right)+\Xi_{3}\left(A,B\right)\right|\right], & \;\text{if}\;\;\left|k\right|<1-A,\\ \frac{\vartheta_{1}^{4}{\left(A-B\right)}^{2}}{4r^{2}}\left[\frac{\Xi_{4}^{2}\left(A,B\right)}{\vartheta_{3}^{2}{\left(\vartheta_{3}-\vartheta_{1}\right)}^{2}}+\frac{1}{\vartheta_{4}\vartheta_{2}\left(\vartheta_{4}-\vartheta_{1}\right)\left(\vartheta_{2}-\vartheta_{1}\right)}\left|\Xi_{1}\left(A,B\right)+\Xi_{2}\left(A,B\right)+\Xi_{3}\left(A,B\right)\right|\right], & \;\text{if}\;\left|k\right|\geq 1-A, \end{array}\right.$$

where

$$\begin{array}{ccc} & & \Xi_{1}\left(A,B\right):=\frac{\vartheta_{1}^{4}\left(\vartheta_{4}-\vartheta_{1}\right){\left(A-B\right)}^{2}}{3\vartheta_{2}^{2}{\left(\vartheta_{2}-\vartheta_{1}\right)}^{3}}-\frac{\vartheta_{1}\left(\vartheta_{3}+\vartheta_{2}-2\vartheta_{1}\right)B\left(A-B\right)}{\left(\vartheta_{3}-\vartheta_{1}\right)\left(\vartheta_{2}-\vartheta_{1}\right)},\\ & & \Xi_{2}\left(A,B\right):=\frac{\vartheta_{1}^{2}\vartheta_{4}\left(\vartheta_{4}-\vartheta_{1}\right)\left(\vartheta_{2}B-\vartheta_{1}A\right)\left(A-B\right)}{\vartheta_{3}\vartheta_{2}\left(\vartheta_{3}-\vartheta_{1}\right){\left(\vartheta_{2}-\vartheta_{1}\right)}^{2}},\\ & & \Xi_{3}\left(A,B\right):=\frac{\vartheta_{1}^{2}{\left(A-B\right)}^{2}+\left(\vartheta_{2}-\vartheta_{1}\right)\left(\vartheta_{3}-\vartheta_{1}\right)B^{2}}{\left(\vartheta_{2}-\vartheta_{1}\right)\left(\vartheta_{3}-\vartheta_{1}\right)},\\ & & \Xi_{4}\left(A,B\right):=\frac{\vartheta_{1}^{2}\vartheta_{3}\left(\vartheta_{3}-\vartheta_{1}\right)\left(A-B\right)+2\vartheta_{2}^{2}\left(\vartheta_{2}-\vartheta_{1}\right)\left(\vartheta_{2}B-\vartheta_{1}A\right)}{2\vartheta_{2}^{2}{\left(\vartheta_{2}-\vartheta_{1}\right)}^{2}}, \end{array}$$

$\rho_{6}$ is defined by (6.2), $\delta_{6}$ is defined by (6.3) and k is defined by (6.1). If we set Φ(z) as defined in (2.20), then Theorems 4.2, 4.4 and 4.6 guide us to the subsequent corollaries:

Corollary 6.4 *Let*$u\in S_{q}^{C}\left(r,\alpha \right)$*be of the form*(2.8)*. Then*

$$\left|T_{2,1}\left(\gamma_{u}\right)\right|\leq \left\{ \begin{array}{cc} \frac{\vartheta_{1}^{4}{\left(1-\alpha \right)}^{2}}{r^{2}}\left[\frac{1}{\vartheta_{3}^{2}{\left(\vartheta_{3}-\vartheta_{1}\right)}^{2}}+\frac{1}{\vartheta_{2}^{2}{\left(\vartheta_{2}-\vartheta_{1}\right)}^{2}}\right], & \;\text{if}\;\left|l\right|<\alpha ,\\ \frac{\vartheta_{1}^{4}{\left(1-\alpha \right)}^{2}}{r^{2}\vartheta_{3}^{2}{\left(\vartheta_{3}-\vartheta_{1}\right)}^{2}}\left[1+{\left(\frac{\vartheta_{1}^{2}\vartheta_{3}\left(\vartheta_{3}-\vartheta_{1}\right)\left(1-\alpha \right)}{\vartheta_{2}^{2}{\left(\vartheta_{2}-\vartheta_{1}\right)}^{2}}-\frac{\vartheta_{2}+\vartheta_{1}-2\vartheta_{1}\alpha }{\vartheta_{2}-\vartheta_{1}}\right)}^{2}\right], & \;\text{if}\;\left|l\right|\geq \alpha , \end{array}\right.$$

where

(6.4) $$l≔\left(\frac{\vartheta_{1}^{2}\vartheta_{3}\left(\vartheta_{3}-\vartheta_{1}\right)}{2\vartheta_{2}^{2}{\left(\vartheta_{2}-\vartheta_{1}\right)}^{2}}-\frac{\vartheta_{2}}{\vartheta_{2}-\vartheta_{1}}\right)\left(1-\alpha \right).$$

Corollary 6.5 *Let*$u\in S_{q}^{C}\left(r,\alpha \right)$*be of the form*(2.8)*and*$\left(\rho_{7},\delta_{7}\right)\in \cup_{i=1}^{5}D_{i}$*holds. Then*

$$\left|T_{2,2}\left(\gamma_{u}\right)\right|\leq \left\{ \begin{array}{cc} \frac{\vartheta_{1}^{4}{\left(1-\alpha \right)}^{2}}{r^{2}\vartheta_{3}^{2}{\left(\vartheta_{3}-\vartheta_{1}\right)}^{2}}\left[1+\frac{1}{\vartheta_{4}^{2}{\left(\vartheta_{4}-\vartheta_{1}\right)}^{2}}{\left(\varphi_{1}\left(\alpha \right)+\varphi_{2}\left(\alpha \right)\right)}^{2}\right], & \;\text{if}\;\left|l\right|<\alpha ,\\ \frac{\vartheta_{1}^{4}{\left(1-\alpha \right)}^{2}}{r^{2}\vartheta_{3}^{2}{\left(\vartheta_{3}-\vartheta_{1}\right)}^{2}}\left[\varphi_{3}^{2}\left(\alpha \right)+\frac{1}{\vartheta_{4}^{2}{\left(\vartheta_{4}-\vartheta_{1}\right)}^{2}}{\left(\varphi_{1}\left(\alpha \right)+\varphi_{2}\left(\alpha \right)\right)}^{2}\right], & \;\text{if}\;\left|l\right|\geq \alpha , \end{array}\right.$$

where

$$\begin{array}{ccc} \varphi_{1}\left(\alpha \right) & := & \frac{4\vartheta_{1}^{4}\vartheta_{3}\left(\vartheta_{4}-\vartheta_{1}\right)\left(\vartheta_{3}-\vartheta_{1}\right){\left(1-\alpha \right)}^{2}}{3\vartheta_{2}^{2}{\left(\vartheta_{2}-\vartheta_{1}\right)}^{3}}+\frac{2\vartheta_{1}\vartheta_{3}\left(\vartheta_{3}+\vartheta_{2}-2\vartheta_{1}\right)\left(1-\alpha \right)}{\vartheta_{2}-\vartheta_{1}},\\ \varphi_{2}\left(\alpha \right) & := & \frac{4\vartheta_{1}\vartheta_{3}{\left(1-\alpha \right)}^{2}+\vartheta_{2}-\vartheta_{1}}{\vartheta_{2}-\vartheta_{1}}-\frac{2\vartheta_{1}^{2}\vartheta_{4}\left(\vartheta_{4}-\vartheta_{1}\right)\left(\vartheta_{2}+\vartheta_{1}-2\vartheta_{1}\alpha \right)\left(1-\alpha \right)}{\vartheta_{2}{\left(\vartheta_{2}-\vartheta_{1}\right)}^{2}},\\ \varphi_{3}\left(\alpha \right) & := & \frac{\vartheta_{1}\left(\vartheta_{1}\vartheta_{3}\left(\vartheta_{3}-\vartheta_{1}\right)-2\vartheta_{2}^{2}\left(\vartheta_{2}-\vartheta_{1}\right)\right)\left(1-\alpha \right)-\vartheta_{2}^{2}{\left(\vartheta_{2}-\vartheta_{1}\right)}^{2}}{\vartheta_{2}^{2}{\left(\vartheta_{2}-\vartheta_{1}\right)}^{2}}, \end{array}$$

(6.5) $$\rho_{7}≔\frac{2\vartheta_{1}\left(\vartheta_{3}+\vartheta_{2}-2\vartheta_{1}\right)\left(1-\alpha \right)}{\left(\vartheta_{3}-\vartheta_{1}\right)\left(\vartheta_{2}-\vartheta_{1}\right)}-\frac{2\vartheta_{1}^{4}\vartheta_{4}\left(\vartheta_{4}-\vartheta_{1}\right)\left(1-\alpha \right)}{\vartheta_{3}\vartheta_{2}\left(\vartheta_{3}-\vartheta_{1}\right)\left(\vartheta_{2}-\vartheta_{1}\right)}+2,$$

(6.6) $$\begin{array}{ccc} \delta_{7} & ≔ & \frac{4\vartheta_{1}^{4}\left(\vartheta_{4}-\vartheta_{1}\right){\left(1-\alpha \right)}^{2}}{3\vartheta_{2}^{2}{\left(\vartheta_{2}-\vartheta_{1}\right)}^{3}}-\frac{2\vartheta_{1}^{2}\vartheta_{4}\left(\vartheta_{4}-\vartheta_{2}\right)}{\vartheta_{3}\vartheta_{2}\left(\vartheta_{3}-\vartheta_{1}\right)\left(\vartheta_{2}-\vartheta_{1}\right)}\left(\frac{2\vartheta_{1}{\left(1-\alpha \right)}^{2}}{\vartheta_{2}-\vartheta_{1}}+1-\alpha \right)+\frac{4\vartheta_{1}^{2}{\left(1-\alpha \right)}^{2}}{{\left(\vartheta_{2}-\vartheta_{1}\right)}^{2}}\left(\frac{\vartheta_{3}+\vartheta_{2}-2\vartheta_{1}}{\vartheta_{3}-\vartheta_{1}}-1\right)\\ & & -\;\frac{2\vartheta_{1}\left(\vartheta_{3}+\vartheta_{2}-2\vartheta_{1}\right)\left(1-\alpha \right)}{\left(\vartheta_{3}-\vartheta_{1}\right)\left(\vartheta_{2}-\vartheta_{1}\right)}+1, \end{array}$$

and l is defined by (6.4).

Corollary 6.6 *Let*$u\in S_{q}^{C}\left(r,\alpha \right)$*be of the form*(2.8)*and*$\left(\rho_{7},\delta_{7}\right)\in \cup_{i=1}^{5}D_{i}$*holds. Then*

$$\left|H_{2,1}\left(\gamma_{u}\right)\right|\leq \left\{ \begin{array}{cc} \frac{\vartheta_{1}^{4}{\left(1-\alpha \right)}^{2}}{r^{2}}\left[\frac{1}{\vartheta_{3}^{2}{\left(\vartheta_{3}-\vartheta_{1}\right)}^{2}}+\frac{1}{\vartheta_{4}\vartheta_{2}\left(\vartheta_{4}-\vartheta_{1}\right)\left(\vartheta_{2}-\vartheta_{1}\right)}\left|\Xi_{1}\left(\alpha \right)-\Xi_{2}\left(\alpha \right)+\Xi_{3}\left(\alpha \right)\right|\right],\;\text{if}\;\;\left|l\right|<\alpha , & \;\\ \frac{\vartheta_{1}^{4}{\left(1-\alpha \right)}^{2}}{r^{2}}\left[\frac{\Xi_{4}^{2}\left(\alpha \right)}{\vartheta_{3}^{2}{\left(\vartheta_{3}-\vartheta_{1}\right)}^{2}}+\frac{1}{\vartheta_{4}\vartheta_{2}\left(\vartheta_{4}-\vartheta_{1}\right)\left(\vartheta_{2}-\vartheta_{1}\right)}\left|\Xi_{1}\left(\alpha \right)-\Xi_{2}\left(\alpha \right)+\Xi_{3}\left(\alpha \right)\right|\right],\;\;\text{if}\;\;\left|l\right|\geq \alpha , \end{array}\right.$$

where

$$\begin{array}{ccc} & & \Xi_{1}\left(\alpha \right):=\frac{4\vartheta_{1}^{4}\left(\vartheta_{4}-\vartheta_{1}\right){\left(1-\alpha \right)}^{2}}{3\vartheta_{2}^{2}{\left(\vartheta_{2}-\vartheta_{1}\right)}^{3}}+\frac{2\vartheta_{1}\left(\vartheta_{3}+\vartheta_{2}-2\vartheta_{1}\right)\left(1-\alpha \right)}{\left(\vartheta_{3}-\vartheta_{1}\right)\left(\vartheta_{2}-\vartheta_{1}\right)},\\ & & \Xi_{2}\left(\alpha \right):=\frac{2\vartheta_{1}^{2}\vartheta_{4}\left(\vartheta_{4}-\vartheta_{1}\right)\left(\vartheta_{2}+\vartheta_{1}-2\vartheta_{1}\alpha \right)\left(1-\alpha \right)}{\vartheta_{3}\vartheta_{2}\left(\vartheta_{3}-\vartheta_{1}\right){\left(\vartheta_{2}-\vartheta_{1}\right)}^{2}},\\ & & \Xi_{3}\left(\alpha \right):=\frac{4\vartheta_{1}^{2}{\left(1-\alpha \right)}^{2}+\left(\vartheta_{2}-\vartheta_{1}\right)\left(\vartheta_{3}-\vartheta_{1}\right)}{\left(\vartheta_{2}-\vartheta_{1}\right)\left(\vartheta_{3}-\vartheta_{1}\right)},\\ & & \Xi_{4}\left(\alpha \right):=\frac{\vartheta_{1}^{2}\vartheta_{3}\left(\vartheta_{3}-\vartheta_{1}\right)\left(1-\alpha \right)}{\vartheta_{2}^{2}{\left(\vartheta_{2}-\vartheta_{1}\right)}^{2}}-\frac{\vartheta_{2}+\vartheta_{1}-2\vartheta_{1}\alpha }{\vartheta_{2}-\vartheta_{1}}, \end{array}$$

$\rho_{7}$ is defined by (6.5), $\delta_{7}$ is defined by (6.6) and l is defined by (6.4). If we define Φ(z) as given in (2.23), then Theorems 4.2, 4.4 and 4.6 direct us to the ensuing corollaries:

Corollary 6.7 *Let*$u\in S_{q}^{C}\left(r,\beta \right)$*be of the form*(2.8)*. Then*

$$\left|T_{2,1}\left(\gamma_{u}\right)\right|\leq \left\{ \begin{array}{cc} \frac{\vartheta_{1}^{4}\beta^{2}}{r^{2}}\left[\frac{1}{\vartheta_{3}^{2}{\left(\vartheta_{3}-\vartheta_{1}\right)}^{2}}+\frac{1}{\vartheta_{2}^{2}{\left(\vartheta_{2}-\vartheta_{1}\right)}^{2}}\right], & \;\text{if}\;\left|m\right|<1-\beta ,\\ \frac{\vartheta_{1}^{4}\beta^{2}}{r^{2}\vartheta_{3}^{2}{\left(\vartheta_{3}-\vartheta_{1}\right)}^{2}}\left[1+{\left(\frac{\vartheta_{1}^{2}\vartheta_{3}\left(\vartheta_{3}-\vartheta_{1}\right)\beta }{\vartheta_{2}^{2}{\left(\vartheta_{2}-\vartheta_{1}\right)}^{2}}-\frac{\left(\vartheta_{2}+\vartheta_{1}\right)\beta }{\vartheta_{2}-\vartheta_{1}}\right)}^{2}\right], & \;\text{if}\;\left|m\right|\geq 1-\beta , \end{array}\right.$$

where

(6.7) $$m≔\left(\frac{\vartheta_{1}^{2}\vartheta_{3}\left(\vartheta_{3}-\vartheta_{1}\right)}{\vartheta_{2}^{2}{\left(\vartheta_{2}-\vartheta_{1}\right)}^{2}}-\frac{2\vartheta_{2}}{\vartheta_{2}-\vartheta_{1}}\right)\beta .$$

Corollary 6.8 *Let*$u\in S_{q}^{C}\left(r,\beta \right)$*be of the form*(2.8)*and*$\left(\rho_{8},\delta_{8}\right)\in \cup_{i=1}^{5}D_{i}$*holds. Then*

$$\left|T_{2,2}\left(\gamma_{u}\right)\right|\leq \left\{ \begin{array}{cc} \frac{\vartheta_{1}^{4}\beta^{2}}{r^{2}\vartheta_{3}^{2}{\left(\vartheta_{3}-\vartheta_{1}\right)}^{2}}\left[1+\frac{1}{\vartheta_{4}^{2}{\left(\vartheta_{4}-\vartheta_{1}\right)}^{2}}{\left(\varphi_{1}\left(\beta \right)+\varphi_{2}\left(\beta \right)\right)}^{2}\right], & \;\text{if}\;\left|m\right|<1-\beta ,\\ \frac{\vartheta_{1}^{4}\beta^{2}}{r^{2}\vartheta_{3}^{2}{\left(\vartheta_{3}-\vartheta_{1}\right)}^{2}}\left[\varphi_{3}^{2}\left(\beta \right)+\frac{1}{\vartheta_{4}^{2}{\left(\vartheta_{4}-\vartheta_{1}\right)}^{2}}{\left(\varphi_{1}\left(\beta \right)+\varphi_{2}\left(\beta \right)\right)}^{2}\right], & \;\text{if}\;\left|m\right|\geq 1-\beta , \end{array}\right.$$

where

$$\begin{array}{ccc} & & \varphi_{1}\left(\beta \right):=\frac{4\vartheta_{1}^{4}\vartheta_{3}\left(\vartheta_{4}-\vartheta_{1}\right)\left(\vartheta_{3}-\vartheta_{1}\right)\beta^{2}}{3\vartheta_{2}^{2}{\left(\vartheta_{2}-\vartheta_{1}\right)}^{3}}+\frac{2\vartheta_{1}\vartheta_{3}\left(\vartheta_{3}+\vartheta_{2}-2\vartheta_{1}\right)\beta^{2}}{\vartheta_{2}-\vartheta_{1}},\\ & & \varphi_{2}\left(\beta \right):=\frac{\left(4\vartheta_{1}\vartheta_{3}+\vartheta_{2}-\vartheta_{1}\right)\beta^{2}}{\vartheta_{2}-\vartheta_{1}}-\frac{2\vartheta_{1}^{2}\vartheta_{4}\left(\vartheta_{4}-\vartheta_{1}\right)\left(\vartheta_{2}+\vartheta_{1}\right)\beta^{2}}{\vartheta_{2}{\left(\vartheta_{2}-\vartheta_{1}\right)}^{2}},\\ & & \varphi_{3}\left(\beta \right):=\frac{\vartheta_{1}^{2}\vartheta_{3}\left(\vartheta_{3}-\vartheta_{1}\right)\beta }{\vartheta_{2}^{2}{\left(\vartheta_{2}-\vartheta_{1}\right)}^{2}}-\frac{\left(\vartheta_{2}+\vartheta_{1}\right)\beta }{\vartheta_{2}-\vartheta_{1}}, \end{array}$$

(6.8) $$\rho_{8}≔\frac{2\vartheta_{1}\left(\vartheta_{3}+\vartheta_{2}-2\vartheta_{1}\right)\beta }{\left(\vartheta_{3}-\vartheta_{1}\right)\left(\vartheta_{2}-\vartheta_{1}\right)}-\frac{2\vartheta_{1}^{4}\vartheta_{4}\left(\vartheta_{4}-\vartheta_{1}\right)\beta }{\vartheta_{3}\vartheta_{2}\left(\vartheta_{3}-\vartheta_{1}\right)\left(\vartheta_{2}-\vartheta_{1}\right)}+2\beta ,$$

(6.9) $$\delta_{8}≔\frac{4\vartheta_{1}^{4}\left(\vartheta_{4}-\vartheta_{1}\right)\beta^{2}}{3\vartheta_{2}^{2}{\left(\vartheta_{2}-\vartheta_{1}\right)}^{3}}-\frac{2\vartheta_{1}^{2}\vartheta_{4}\left(\vartheta_{4}-\vartheta_{1}\right)\beta^{2}}{\vartheta_{3}\vartheta_{2}\left(\vartheta_{3}-\vartheta_{1}\right)\left(\vartheta_{2}-\vartheta_{1}\right)}\left(\frac{2\vartheta_{1}}{\vartheta_{2}-\vartheta_{1}}+1\right)+\frac{4\vartheta_{1}^{2}\beta^{2}}{{\left(\vartheta_{2}-\vartheta_{1}\right)}^{2}}\left(\frac{\vartheta_{3}+\vartheta_{2}-2\vartheta_{1}}{\vartheta_{3}-\vartheta_{1}}-1\right)-\frac{2\vartheta_{1}\left(\vartheta_{3}+\vartheta_{2}-2\vartheta_{1}\right)\beta^{2}}{\left(\vartheta_{3}-\vartheta_{1}\right)\left(\vartheta_{2}-\vartheta_{1}\right)}+\beta^{2},$$

and m is defined by (6.7).

Corollary 6.9 *Let*$u\in S_{q}^{C}\left(r,\beta \right)$*be of the form*(2.8)*and*$\left(\rho_{8},\delta_{8}\right)\in \cup_{i=1}^{5}D_{i}$*holds. Then*

$$\left|H_{2,1}\left(\gamma_{u}\right)\right|\leq \left\{ \begin{array}{cc} \frac{\vartheta_{1}^{4}\beta^{2}}{r^{2}}\left[\frac{1}{\vartheta_{3}^{2}{\left(\vartheta_{3}-\vartheta_{1}\right)}^{2}}+\frac{1}{\vartheta_{4}\vartheta_{2}\left(\vartheta_{4}-\vartheta_{1}\right)\left(\vartheta_{2}-\vartheta_{1}\right)}\left|\Xi_{1}\left(\beta \right)-\Xi_{2}\left(\beta \right)+\Xi_{3}\left(\beta \right)\right|\right],\;\text{if}\;\;\left|m\right|<1-\beta , & \;\\ \frac{\vartheta_{1}^{4}\beta^{2}}{r^{2}}\left[\frac{\Xi_{4}^{2}\left(\beta \right)}{\vartheta_{3}^{2}{\left(\vartheta_{3}-\vartheta_{1}\right)}^{2}}+\frac{1}{\vartheta_{4}\vartheta_{2}\left(\vartheta_{4}-\vartheta_{1}\right)\left(\vartheta_{2}-\vartheta_{1}\right)}\left|\Xi_{1}\left(\beta \right)-\Xi_{2}\left(\beta \right)+\Xi_{3}\left(\beta \right)\right|\right],\;\;\text{if}\;\left|m\right|\geq 1-\beta , \end{array}\right.$$

where

$$\begin{array}{ccc} & & \Xi_{1}\left(\beta \right):=\frac{4\vartheta_{1}^{4}\left(\vartheta_{4}-\vartheta_{1}\right)\beta^{2}}{3\vartheta_{2}^{2}{\left(\vartheta_{2}-\vartheta_{1}\right)}^{3}}+\frac{\vartheta_{1}\left(\vartheta_{3}+\vartheta_{2}-2\vartheta_{1}\right)\beta^{2}}{\left(\vartheta_{3}-\vartheta_{1}\right)\left(\vartheta_{2}-\vartheta_{1}\right)},\\ & & \Xi_{2}\left(\beta \right):=\frac{\vartheta_{1}^{2}\vartheta_{4}\left(\vartheta_{4}-\vartheta_{1}\right)\left(\vartheta_{2}+\vartheta_{1}\right)\beta^{2}}{\vartheta_{3}\vartheta_{2}\left(\vartheta_{3}-\vartheta_{1}\right){\left(\vartheta_{2}-\vartheta_{1}\right)}^{2}},\\ & & \Xi_{3}\left(\beta \right):=\frac{\left(4\vartheta_{1}^{2}+\left(\vartheta_{2}-\vartheta_{1}\right)\left(\vartheta_{3}-\vartheta_{1}\right)\right)\beta^{2}}{\left(\vartheta_{2}-\vartheta_{1}\right)\left(\vartheta_{3}-\vartheta_{1}\right)},\\ & & \Xi_{4}\left(\beta \right):=\frac{\left(\vartheta_{1}^{2}\vartheta_{3}\left(\vartheta_{3}-\vartheta_{1}\right)-\vartheta_{2}^{2}\left(\vartheta_{2}^{2}-\vartheta_{1}^{2}\right)\right)\beta }{\vartheta_{2}^{2}{\left(\vartheta_{2}-\vartheta_{1}\right)}^{2}}, \end{array}$$

$\rho_{8}$ is defined by (6.8), $\delta_{8}$ is defined by (6.9) and m is defined by (6.7).

## Concluding remarks and observations

Our current study is driven by the established opportunities presented by the applications of q-calculus and fractional q-calculus in GFT, as detailed in a recently published review article by Srivastava [50]. In this paper, we have systematically introduced and investigated compelling subfamilies of r-valent q-starlike and multivalent q-convex functions within D. Our exploration has centered on addressing intriguing problems that contribute to a better understanding of the geometry of the image domain. Moreover, we have harnessed some of our obtained results to analyze the growth of Toeplitz and Hankel determinants. These determinants involve the first, second, and third logarithmic coefficients of functions belonging to the multivalent q-starlike and multivalent q-convex function families.

The concept investigated in this article can readily be applied to define numerous subfamilies of holomorphic and univalent (or multivalent) functions that are associated with diverse image domains, such as cardioid and lemniscate domains. This, in turn, paves the way for numerous research opportunities in special functions, harmonic univalent functions, uniformly starlike functions, uniformly convex functions and their fuzzy aspects, particularly in the study of analytic and meromorphic function families shaped by quantum calculus and fractional operators. In fact, it becomes possible to investigate the bounds of Hankel and Toeplitz determinants, expressed as follows:

$$H_{2,2}\left(\gamma_{u}\right)=\gamma_{2}\gamma_{4}-\gamma_{3}^{2},T_{2,3}\left(\gamma_{u}\right)=\gamma_{3}^{2}-\gamma_{4}^{2}\;\text{and}\;T_{3,2}\left(\gamma_{u}\right)=\gamma_{2}^{3}+2\gamma_{3}^{2}\left(\gamma_{4}-\gamma_{2}\right)-\gamma_{2}\gamma_{4}^{2}$$

where $\gamma_{2}$ and $\gamma_{3}$ are given by (2.11) and (2.12), respectively, and

$$\gamma_{4}=\frac{1}{2r}\left(a_{r+4}-a_{r+1}a_{r+3}+a_{r+1}^{2}a_{r+2}-\frac{1}{2}a_{r+2}^{2}-\frac{1}{4}a_{r+1}^{4}\right).$$

## Method validation

Not applicable.

## Limitations

None.

## Ethical approval

Not applicable.

## CRediT author statement

Both authors made equal contributions to this work and approved the final version of the manuscript.

## Declaration of competing interest

The authors declare that they have no known competing financial interests or personal relationships that could have appeared to influence the work reported in this paper.

## Data Availability

No data was used for the research described in the article.
